# Synthesis of Schiff Bases Containing Phenol Rings and Investigation of Their Antioxidant Capacity, Anticholinesterase, Butyrylcholinesterase, and Carbonic Anhydrase Inhibition Properties

**DOI:** 10.3390/pharmaceutics15030779

**Published:** 2023-02-26

**Authors:** Sertan Aytac, Ozlem Gundogdu, Zeynebe Bingol, İlhami Gulcin

**Affiliations:** 1 Department of Food Technology, Kaman Technical and Vocational School, Kirsehir Ahi Evran University, 40100 Kırşehir, Turkey; 2 Vocational School of Health Services, Gaziosmanpasa University, 60250 Tokat, Turkey; 3 Department of Chemistry, Faculty of Science, Atatürk University, 25240 Erzurum, Turkey

**Keywords:** Schiff bases, antioxidant activity, enzyme inhibition, acetylcholinesterase, microwave irradiation, butyrylcholinesterase

## Abstract

The widespread usage of Schiff bases in chemistry, industry, medicine, and pharmacy has increased interest in these compounds. Schiff bases and derivative compounds have important bioactive properties. Heterocyclic compounds containing phenol derivative groups in their structure have the potential to capture free radicals that can cause diseases. In this study, we designed and synthesized eight Schiff bases (**10**–**15**) and hydrazineylidene derivatives (**16**–**17**), which contain phenol moieties and have the potential to be used as synthetic antioxidants, for the first time using microwave energy. Additionally, the antioxidant effects of Schiff bases (**10**–**15**) and hydrazineylidene derivatives (**16**–**17**) were studied using by the bioanalytical methods of 2,2’-azino-bis(3-ethylbenzothiazoline-6-sulfonic acid) cation radical (ABTS^•+^) and 1,1-diphenyl-2-picrylhydrazyl (DPPH^•^) scavenging activities, and Fe^3+^, Cu^2+^, and Fe^3+^-TPTZ complex reducing capacities. In the context of studies on antioxidants, Schiff bases (**10**–**15**) and hydrazineylidene derivatives (**16**–**17**) were found to be as powerful DPPH (IC_50_: 12.15–99.01 μg/mL) and ABTS^•+^ (IC_50_: 4.30–34.65 μg/mL). Additionally, the inhibition abilities of Schiff bases (**10**–**15**) and hydrazineylidene derivatives (**16**–**17**) were determined towards some metabolic enzymes including acetylcholinesterase (AChE), butyrylcholinesterase (BChE), and human carbonic anhydrase I and II (hCAs I and II), enzymes that are linked to some global disorders including Alzheimer’s disease (AD), epilepsy, and glaucoma. In the context of studies on enzyme inhibition, it was observed that the synthesized Schiff bases (**10**–**15**) and hydrazineylidene derivatives (**16**–**17**) inhibited AChE, BChE, hCAs I, and hCA II enzymes with IC_50_ values in ranges of 16.11–57.75 nM, 19.80–53.31 nM, 26.08 ± 8.53 nM, and 85.79 ± 24.80 nM, respectively. In addition, in light of the results obtained, we hope that this study will be useful and guiding for the evaluation of biological activities in the fields of the food, medical, and pharmaceutical industries in the future.

## 1. Introduction

Excessive formation of reactive oxygen species (ROS) and free radicals in living metabolism can cause cell death by damaging many cellular biomolecules, especially nucleic acids, membrane lipids, and proteins. On the other hand, antioxidants neutralize free radicals and effectively terminate radical chain reactions [1,2]. Hydroxyl radicals (OH‧), superoxide anion radicals (O_2_‧^−^), hydrogen peroxide (H_2_O_2_), and singlet oxygen (^1^O_2_) can be considered the most common ROS. They are formed as a result of normal metabolic functions of the body or as a result of physical and mental stress. Additionally, radiations, organic solvents, pesticides, and cigarette smoke are thought to be exogenous sources of ROS and free radical sources [3].

Antioxidants protect living organisms from hazardous effects of ROS by reducing the generation of free radicals. In addition, ROS and free radicals have been reported to be responsible for many global diseases including cancer, atherosclerosis, rheumatoid arthritis, neurodegenerative, autoimmune, cardiovascular, and age-related diseases [4]. In this regard, it is critical to avoid or delay the development of ROS that cause a various of health problems. In living organisms, antioxidant defense mechanisms prevent the formation of ROS and reduce their cellular damage [5]. Nowadays, the most commonly used synthetic antioxidants are butylated hydroxyanisole (BHA), *tert*-butylhydroquinone (TBHQ), butylated hydroxytoluene (BHT), and propyl gallate (PG). They are added to food products during processing, thereby increasing the resistance of food to degradation and extending its shelf life. However, it has been reported that there are findings about serious health problems of synthetic antioxidants such as liver toxicity [6,7]. Therefore, this situation has recently led researchers to the synthesis of more alternative and safer antioxidant compounds.

In previous studies, it has been reported that antioxidant molecules inhibit acetylcholinesterase (AChE), butyrylcholinesterase (BChE), and carbonic anhydrase (CA) enzymes, which are linked to some common diseases including epilepsy, glaucoma, and Alzheimer’s disease (AD) [8]. Recently, Schiff bases and their derivatives have attracted the attention of researchers [9,10]. It is well-known that ROS and free radicals alter the structure of human cells, causing various diseases such as cancer, neurological problems, AD, and diabetes. Of these, AD is a common neurological disease that progresses with rapid behavioral change with memory loss, forgetfulness, and impaired cognition and language [11,12,13,14]. An important side effect of AD pathology is the lack of cholinergic neurons in the brain of AD patients [15,16]. Therefore, the activation of cholinergic receptors is an attractive and highly important therapeutic possibility for AD. This option can be achieved by inhibiting the ACh degradation using AChE inhibitors (AChEIs) such as donepezil, tacrine, and rivastigmine, which are approved for the treatment of AD [17]. It is well-known that most AChEIs have undesired side effects such as gastrointestinal anomalies, diarrhea, nausea, and hepatotoxicity. Therefore, Tacrine is no longer used due to the emergence of hepatotoxicity effects [18]. AChEIs are the most-prescribed drug classes for AD treatment. Therefore, the search for new and undesired side effect-free cholinesterase inhibitors has become a necessity in the pharmaceutical field [19,20].

Carbonic anhydrases (CAs, E.C.4.2.1.1) catalyze the conversion between CO_2_ and HCO_3_^−^ for generating a H^+^ and pH regulating. To date, eight distinct CA families (α, β, γ, δ, ζ, η, θ, and t-CAs) are known. Of these, humans only have α-CAs, which have sixteen CA isoforms [21]. These isoenzymes differ in cellular localization, oligomeric structure, distribution in organs and tissues, affinity for inhibition, kinetic and molecular properties, and expression levels. CA inhibitors (CAIs) have been designed to obtain diuretic, antiglaucoma, antiepileptic, and antitumor agents. They have drug potential for many biochemical and pharmacological applications [22,23]. CA II isozyme inhibition is associated with a variety of tumors, including kidney, lung, and esophageal cancers, melanoma, and glaucoma disease [24,25].

**Figure 1:**
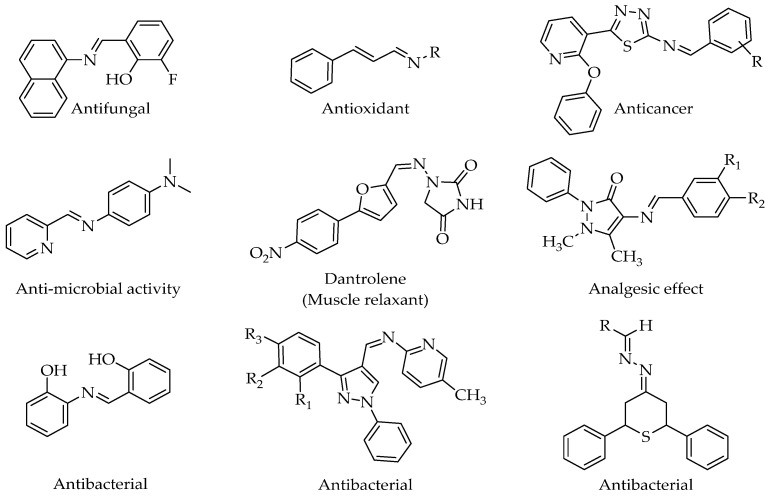
Chemical structures of some pharmacologically active Schiff bases.

Heterocyclic compounds containing nitrogen atoms have various biological properties. Schiff bases as a crucial class of heterocyclic compounds show anticonvulsant, antidepressant, anti-inflammatory, analgesic, antimicrobial, antimalarial, anticancer, and antioxidant activities [26,27]. These chemicals are significant in the synthesis of many physiologically and medically active substances. Schiff bases and derivatives are also known as important intermediates for the synthesis of N-containing heterocyclic substances. They are commonly represented by the R-CH=N-Ar formula [28,29]. Schiff bases are commonly found in the structure of naturally occurring products. They have an important role in synthesis and pharmaceutical research. The chemical structures of some pharmacologically active Schiff bases are given in Figure 1.

Imine structures have drug potential for diseases caused by free radical damage due to their antioxidant abilities [30]. When the literature is reviewed, it is seen that Schiff bases exhibit antioxidant ability by removing ROS [31]. Additionally, the connection between the chemical structures of Schiff bases and their antioxidant ability is well established [32]. Additionally, Schiff bases and derivatives containing the azomethine group (–N=CH–) have different applications. Therefore, they are of great interest due to their potential biological properties, including their antioxidant effects.

Heterocyclic structures containing imine and phenol groups have ROS and free radical scavenging abilities. Therefore, they have potential to be used as a medicine against some diseases induced by oxidative stress [30]. Among the chemicals that can be utilized as synthetic antioxidants, Schiff bases have a significant role and potentials. By reviewing the literature, we observed that many studies have been carried out on the antioxidant ability of Schiff bases. They are conventionally obtained through the heat treatment of ketones or aldehydes with amine compounds under acidic conditions. With the latest developments in chemistry, alternative assays have been searched and utilized for the synthesis of Schiff bases [33]. Scientists have synthesized many new and chemical compounds using the heating method. However, this method generally takes a long time and increases chemical consumption and costs [34]. The use of microwave energy has become an appreciated and popular topic in synthetic organic chemistry. The use of this energy in experiments after the 1980s has brought great benefits in organic synthesis [35]. This technique has many advantages over the classical techniques. It reduces the formation of by-products and the evaporation of solvents, shortens the reaction time, and the reactions result in higher yields [36].

**Scheme 1:**
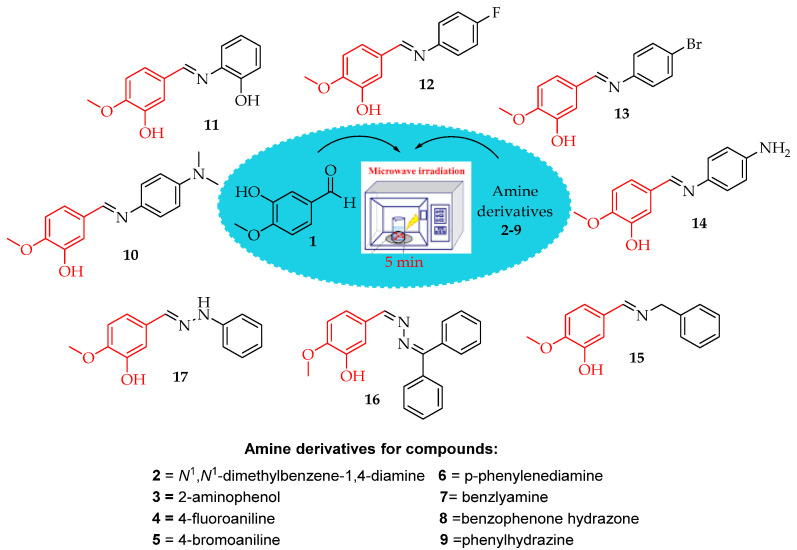
Synthesis method for Schiff bases (**10**–**15**) and hydrazineylidene derivatives (**16** and **17**).

Recently, increasing studies on Schiff bases and the antioxidant activity of these bases encouraged us to carry out this study. In this work, we synthesized eight Schiff bases and hydrazineylidene derivatives (**10**–**17**) containing phenol rings using microwave irradiation (Scheme 1). Another important goal of this study is to examine the antioxidant abilities of these compounds. For this purpose, isovanillin (**1**) and different amine compounds (**2**–**9**) were used as starting compounds for the synthesis of eight different phenol ring-containing Schiff base derivatives (Scheme 1).

Except for compounds **11** and **16**, the compounds were synthesized for the first time. It is known from the literature that compounds **11** and **16** were synthesized [37] through the condensation method using methanol or ethanol at rt or reflux. However, in this study, for the re-synthesis of compound **11** and **16,** the microwave method was also used for the first time. The structures of these compounds were determined using ^1^H-NMR, ^13^C-NMR, FT-IR spectroscopies, and HR-MS. Then, their antioxidant properties were investigated using ABTS^•+^ and DPPH· scavenging abilities, and Fe^3+^, Cu^2+^, and Fe^3+^-TPTZ complex reducing capacities. Additionally, the inhibition effects of Schiff bases (**10**–**15**) and hydrazineylidene derivatives (**16** and **17**) were determined against some metabolic enzymes including AChE, BChE, hCA I, and hCA II enzymes, which are linked to some global disorders including Alzheimer’s disease (AD), epilepsy, and glaucoma.

## 2. Materials and Methods

### 2.1. Chemicals

Acetylcholinesterase, acetylcholine iodide2,2′-azino-bis 3-ethylbenzthiazoline-6-sulfonic acid (ABTS), 1,1-diphenyl-2-picrylhydrazyl (DPPH), N,N-dimethyl-p-phenylenediamine (DMPD), 2,9-dimethyl-1,10-phenanthroline (Neocuproine), butylated hydroxytoluene (BHT), butylated hydroxyanisole (BHA), α-tocopherol, trolox, and solvents were obtained from Sigma-Aldrich (Sigma-Aldrich GmbH, Steinheim, Germany). They were of analytical grade and used without further purification steps.

### 2.2. Materials and Apparatus

The reactions were visualized via thin-layer chromatography (TLC, 60-mesh, Darmstadt, Germany). ^1^H NMR and ^13^C NMR spectra were taken at 400 MHz and 100 MHz using CDCl_3_ (Varian spectrometer, Danbury, CT, USA). The melting points of Schiff bases were determined on a capillary melting apparatus and were uncorrected (BUCHI 530). Reactions were performed via microwave oven (Vestel MD 20 DB model, 230 V-50 Hz, 900 W). HR-MS: electron spray technique (M+/M-) from the soln. in MeOH (Waters LCT Premier^TM^ XE UPLC/MS TOF (Manchester, UK).

### 2.3. General Synthesis of Schiff Bases

Schiff bases **10**–**17** were synthesized as described according to the procedures given in the literature [38,39]. Isovanillin (**1**) (1 mmol) was added to *N*^1^,*N*^1^-dimethylbenzene-1,4-diamine (**2**), 2-aminophenol (**3**), 4-fluoroaniline (**4**), 4-bromoaniline (**5**), p-phenylenediamine (**6**), benzlyamine (**7**), benzophenone hydrazone (**8**), or phenylhydrazine (**9**) (1 mmol). Then, the reaction mixtures were exposed to microwave radiation at 900 W and monitored using TLC. Compounds were purified through crystallization (MeOH/Petroleum ether) or column chromatography (EtOAc/Petroleum ether). For the compounds with (*E*)-configuration, it was observed that a single product (except **14**) formed, according to ^1^H NMR and ^13^C NMR spectrum results.

### 2.4. Physical Properties and Spectral Data of Synthesized Compounds

#### 2.4.1. (*E*)-5-(((4-(Dimethylamino)phenyl)imino)methyl)-2-methoxyphenol (**10**)

It was obtained in a 97% yield as a brown solid. M.p: 147–148 °C. R*_f_*: 0.46 (2:3 EtOAc/Petroleum ether). ^1^H NMR (400 MHz, CDCl^3^) δ 8.41 (s, 1H), 7.54 (d, *J* = 2.0 Hz, 1H), 7.38 (dd, *J* = 8.4, 2.0 Hz, 1H), 7.31–7.24 (m, 2H), 6.91 (d, *J* = 8.4 Hz, 1H), 6.81–6.76 (m, 2H), 3.92 (s, 3H), 2.99 (s, 6H). ^13^C NMR (100 MHz, CDCl_3_) δ 155.85, 149.36, 148.9, 145.98, 141.24, 130.70, 122.17, 121.57, 113.82, 113.09, 110.47, 55.98, 40.85. FT-IR (cm^−1^): 2885 (OH), 1611(C=N), 1514 (C=C), 1438, 1349, 1278. HRMS: (ESI), *m*/*z*: [M + H]+ Calcd for C_16_H_19_N_2_O_2_ 271.1368; found 271.1443.

#### 2.4.2. (*E*)-5-(((2-Hydroxyphenyl)imino)methyl)-2-methoxyphenol (**11**)

It was obtained in a 97% yield as a yellow solid. M.p: 124–125 °C. R*_f_*: 0.53 (2:3 EtOAc/Petroleum ether). ^1^H NMR (400 MHz, CDCl_3_) δ 8.59 (s, 1H), 7.60 (d, *J* = 1.4 Hz, 1H), 7.39 (d, *J* = 8.3 Hz, 1H), 7.29 (d, *J* = 7.3 Hz, 1H), 7.20 (t, *J* = 7.7 Hz, 1H), 7.03 (d, *J* = 7.9 Hz, 1H), 6.93 (dd, *J* = 16.4, 8.1 Hz, 2H), 3.98 (s, 3H). ^13^C NMR (100 MHz, CDCl_3_) δ 156.55, 152.18, 149.70, 146.04, 135.73, 129.83, 128.42, 122.85, 120.04, 115.75, 114.83, 113.38, 110.35, 56.09. FT-IR (cm^−1^): 2939 (OH), 1643(C=N), 1579 (C=C aromatic), 1509, 1438, 1277, 1130, 1026. HRMS: (ESI), *m*/*z*: [M + H]+ Calcd for C_14_H_14_NO_3_ 244.0895; found 244.0960.

#### 2.4.3. (*E*)-5-(((4-Fluorophenyl)imino)methyl)-2-methoxyphenol (**12**)

It was obtained in a 97% yield as a white solid. M.p: 117–118 °C. R*_f_*: 0.57 (2:3 EtOAc/Petroleum ether). ^1^H NMR (400 MHz, CDCl_3_) δ 8.32 (s, 1H), 7.55 (d, *J* = 1.9 Hz, 1H), 7.38 (dd, *J* = 8.3, 1.9 Hz, 1H), 7.25–7.15 (m, 2H), 7.08 (dd, *J* = 11.8, 5.5 Hz, 2H), 6.92 (dd, *J* = 8.3, 2.1 Hz, 1H), 6.14 (s, 1H, OH), 3.93 (s, 3H). ^13^C NMR (100 MHz, CDCl_3_) δ 162.27, 159.85, 159.73, 149.63, 148.19, 146.05, 129.88, 122.39, 122.34, 122.26, 115.89, 115.67, 113.94, 110.42, 55.99. FT-IR (cm^−1^): 2944, 1643 (C=N), 1578 (C=C aromatic), 1501, 1438, 1278, 1223, 1198. HRMS: (ESI), *m*/*z*: [M + H]^+^ Calcd for C_14_H_13_FNO_2_ 246.0852; found 246.0928.

#### 2.4.4. (*E*)-5-(((4-Bromophenyl)imino)methyl)-2-methoxyphenol (**13**)

It was obtained in a 97% yield as a white solid. M.p: 158–160 °C. R*_f_*: 0.60 (2:3 EtOAc/Petroleum ether). ^1^H NMR (400 MHz, CDCl_3_) δ 8.33 (s, 1H), 7.55 (d, *J* = 2.0 Hz, 1H), 7.53–7.48 (m, 2H), 7.39 (dd, *J* = 8.3, 2.0 Hz, 1H), 7.12–7.06 (m, 2H), 6.95 (d, *J* = 8.3 Hz, 1H), 5.72 (s, 1H, OH), 3.99 (s, 3H). ^13^C NMR (100 MHz, CDCl_3_) δ 160.09, 151.19, 149.58, 146.01, 132.13, 129.89, 122.62, 122.52, 118.93, 113.85, 110.33, 56.06. FT-IR (cm^−1^): 2936 (OH), 1611 (C=N), 1576(C=C aromatic), 1512, 1480, 1439, 1277. HRMS: (ESI), *m*/*z*: [M + H]+ Calcd for C_14_H_13_BrNO_2_ 306.0051; found 306.0114.

#### 2.4.5. (E/Z)-5-(((4-Aminophenyl)imino)methyl)-2-methoxyphenol (**14**)

An isomer mixture of (E/Z)-5-(((4-aminophenyl)imino)methyl)-2-methoxyphenol (**14**) was obtained with 97% yield as a yellow solid. M.p: 207–208 °C. R*_f_*: 0.40 (3:2 EtOAc/Petroleum ether). ^1^H NMR (400 MHz, CDCl_3_) δ 8.42 (syn), 8.39 (anti) (s, 2H, CH=N), 7.57, 7.53 (d, *J* = 1.9 Hz, 2H, Ar-H), 7.41, 7.37 (dd, *J* = 8.3, 2.0 Hz, 2H, Ar-H), 7.28 (d, *J* = 3.5 Hz, 2H, NH2), 7.18–7.13 (m, 3H, 2xAr-H and NH), 6.93 (d, *J* = 8.4 Hz, 2H, Ar-H), 6.76–6.71 (m, 3H, 2xAr-H and OH), 6.60 (s, 1H, NH), 3.99 (syn), 3.97 (anti) (s, 6H, 2xOCH_3_). 13C NMR (100 MHz, CDCl_3_) δ 156.50, 149.99, 148.88, 145.88, 144.83, 143.24, 122.27, 121.72, 115.63, 113.62, 110.32, 56.02. FT-IR (cm^−1^): 2971 (OH), 1643 (C=N), 1573 (C=C aromatic), 1510, 1438, 1276, 1251. HRMS: (ESI), *m*/*z*: [M + H]^+^ Calcd for C_14_H_15_N_2_O_2_ 243.1055; found 243.1127.

#### 2.4.6. (*E*)-5-((Benzylimino)methyl)-2-methoxyphenol (**15**)

It was obtained in a 98% yield as a yellow solid. M.p: 97–98 °C. R*_f_*: 0.59 (2:3 EtOAc/Petroleum ether). ^1^H NMR (400 MHz, CDCl_3_) δ 8.29 (s, 1H), 7.44 (d, *J* = 2.0 Hz, 1H), 7.36 (d, *J* = 4.4 Hz, 4H), 7.31–7.26 (m, 2H), 6.89 (d, *J* = 8.3 Hz, 1H), 4.82 (s, 2H), 3.92 (s, 3H). ^13^C NMR (100 MHz, CDCl_3_) δ 161.53, 149.00, 145.92, 139.52, 129.93, 128.46, 127.98, 126.91, 121.33, 113.79, 110.31, 64.83, 55.97. FT-IR (cm^−1^): 2840, 1641(C=N), 1610, 1585 (C=C aromatic), 1512, 1440 (C-N), 1277, 1255, 1132, 1026. HRMS: (ESI), *m*/*z*: [M + H]+ Calcd for C_15_H_16_NO_2_ 242.1103; found 242.1172.

#### 2.4.7. (*E*)-5-(((Diphenylmethylene)hydrazineylidene)methyl)-2-methoxyphenol (**16**)

It was obtained in a 96% yield as an orange liquid. R*_f_*: 0.46 (2:3 EtOAc/Petroleum ether). ^1^H NMR (400 MHz, CDCl_3_) δ 8.54 (s, 1H), 7.79–7.72 (m, 2H), 7.50–7.37 (m, 8H), 7.32 (d, *J* = 1.5 Hz, 1H), 7.19 (dd, *J* = 8.2, 1.5 Hz, 1H), 6.86 (d, *J* = 8.3 Hz, 1H), 5.79 (s, 1H, OH), 3.91 (s, 3H). ^13^C NMR (100 MHz, CDCl_3_) δ 166.11, 159.42, 149.20, 145.86, 138.53, 135.56, 130.46, 130.17, 129.16, 128.96, 128.25, 127.60, 122.23, 113.60, 110.44, 55.96. FT-IR (cm^−1^): 3057 (OH), 1608 (C=N), 1576 (C=C aromatic), 1457, 1442, 1321, 1273, 1132. HRMS: (ESI), *m*/*z*: [M + H]+ Calcd for C_21_H_19_N_2_O_2_ 331.1368; found 331.1436.

#### 2.4.8. (*E*)-2-Methoxy-5-((2-phenylhydrazineylidene)methyl)phenol (**17**)

It was obtained in a 96% yield as a white solid. M.p: 125–127 °C. R*_f_*: 0.61 (2:3 EtOAc/Petroleum ether). ^1^H NMR (400 MHz, CDCl_3_) δ 7.60 (s, 1H), 7.52 (s, 1H, NH), 7.37 (d, *J* = 2.0 Hz, 1H), 7.33–7.26 (m, 2H), 7.11 (dd, *J* = 13.3, 4.8 Hz, 3H), 6.93–6.82 (m, 2H), 5.67 (s, 1H, OH), 3.93 (s, 3H). ^13^C NMR (100 MHz, CDCl_3_) δ 147.15, 145.85, 144.90, 137.22, 129.28, 129.13, 119.85, 119.03, 112.68, 111.63, 110.50, 56.01. FT-IR (cm^−1^): 3021 (OH), 1598 (C=N), 1574 (C=C aromatic), 1506, 1441 (C-N), 1272, 1124. HRMS: (ESI), *m*/*z*: [M + H]+ Calcd for C_14_H_15_N_2_O_2_ 243.1055; found 243.1105.

### 2.5. Reducing Ability Assays

The Fe^3+^ reducing ability of Schiff bases (**10**–**15**) and hydrazineylidene derivatives (**16** and **17**) were tested according to our prior studies [40]. For this purpose, various concentrations of compounds (**10**–**17**) were transferred into test tubes, and 2.5 mL of phosphate buffer (0.2 M, pH 6.6) and 2.5 mL (1%) of potassium ferricyanide [K_3_Fe(CN)_6_] solutions were added. Then, the mixture was vortexed and incubated at 50 °C for 20 min. A portion of trichloroacetic acid (2.5 mL, 10%) was added. Then, 2.5 mL of upper layers of the solutions were mixed with 2.5 mL distilled water and 0.5 mL FeCl_3_ (0.1%). The absorbance values of the reducing effects of compounds (**10**–**17**) and standards were spectrophotometrically recorded at 700 nm.

The Cu^2+^ reducing ability of Schiff bases (**10**–**15**) and hydrazineylidene derivatives (**16** and **17**) were detected according to a prior study [41]. For this purpose, 0.25 mL of CuCl_2_ solution (10 mM), 0.25 mL of ethanolic neocuproine solution (7.5 × 10^−3^ M), and 250 μL of NH_4_Ac buffer solution (1.0 M) in different concentrations (10–30 μg/mL) were transferred to test tubes containing Schiff base (**10**–**15**) and hydrazineylidene derivative (**16** and **17**) samples. The total volume was made up to 2 mL with distilled water, and their absorbance values were recorded at 450 nm after 30 min of incubation.

The Fe^3+^-TPTZ complex reducing ability of Schiff bases (**10**–**15**) and hydrazineylidene derivatives (**16** and **17**) was realized according to a previous study [42]. For this, 2.25 mL of TPTZ solution (10 mM in 40 mM HCl) was freshly prepared, then added to 2.5 mL of acetate buffer (0.3 M, pH 3.6) and 2.25 mL of FeCl_3_ solution (20 mM). Then, different concentrations of Schiff bases (**10**–**15**) and hydrazineylidene derivatives (**16** and **17**) were transferred and incubated at 37 °C for 30 min. Finally, the absorbance values of the reducing power of Schiff bases (**10**–**15**) and hydrazineylidene derivatives (**16** and **17**) and standards were spectrophotometrically measured at 593 nm. All experiments of reducing abilities were repeated three times and the results are given as the arithmetic mean of these repetitions.

### 2.6. Radical Scavenging Capacities

DPPH· and ABTS^•+^ scavenging methods are the most widely used spectrophotometric methods to determine the antioxidant capacity of newly synthesized compounds. The DPPH‧ scavenging effect of Schiff bases (**10**–**15**) and hydrazineylidene derivatives (**16** and **17**) was realized according to the Blois method [43]. Briefly, 1 mL of DPPH^•^ solution (0.1 mM), which was prepared in ethanol and was a violet/purple color depending on the concentration of the antioxidant, was added to the Schiff base (**10**–**15**) and hydrazineylidene derivative (**16** and **17**) samples at different concentrations (10–30 μg/mL). Then, they were incubated at room temperature for 30 min and their absorbance values were recorded at 517 nm.

The ABTS radical cation scavenging assay was used as a way to calculate antioxidant capacity based on this radical scavenging ability. Firstly, an aqueous solution of ABTS (7.0 mM) was oxidized by oxidants such as K_2_S_2_O_8_ (2.5 mM) for the production of its radical cation (ABTS^•+^) [44]. The ABTS^•+^ solution was diluted with a phosphate buffer (0.1 M, pH 7.4) prior to use, adjusting the absorbance value of the control to 0.750 ± 0.025 at 734 nm. Then, 1 mL of ABTS^•+^ solution was added to 3 mL Schiff base (**10**–**15**) and hydrazineylidene derivative (**16** and **17**) solutions at different concentrations (10–30 μg/mL). After 30 min, the remaining absorbance of ABTS^•+^ measured at 734 nm.

### 2.7. AChE and BChE Inhibition Assay

The AChE and BChE inhibition of Schiff bases (**10**–**15**) and hydrazineylidene derivatives (**16** and **17**) was realized according to a putative Ellman’s assay as given in previous studies [45]. Acetylthiocholine iodide/butyrylthiocholine iodide (AChI)/BChI) and 5,5′-dithiobis(2-nitro-benzoic acid) (DTNB) were used as the substrate pattern for both cholinergic reactions. Briefly, 1 mL of Tris/HCl buffer (1.0 M, pH 8.0), 10 μL of different concentrations of Schiff bases (**10**–**15**) and hydrazineylidene derivatives (**16** and **17**), and 50 μL AChE/BChE enzymes were mixed in a test tube. Then, the samples were incubated at 25 °C for 15 min, and 50 μL of DTNB solution (0.5 mM,) was transferred. Then, the reaction was started by adding 50 μL of AChI/BChI solutions (10 mM), and absorbances were recorded at 412 nm. All experiments were repeated three times and the results are given as the arithmetic mean of these repetitions.

### 2.8. Carbonic Anhydrase Purification and Inhibition Studies

Both hCA I and II isoforms were purified using the affinity chromatography technique including Sepharose-4B-L-Tyrosine-sulfanilamide affinity material [46]. Both isoforms’ activity was spectrophotometrically determined according to the Verpoorte assay [47]. One CA unit is defined as the CA quantity, which catalyzes p-Nitrophenylacetate substrate to p-nitrophenolate in 3 min at 348 nm (25 °C). The protein quantity was spectrophotometrically measured at 595 nm according to the Bradford method as bovine serum albumin equivalent [48]. SDS-PAGE was realized according to the Laemmle procedure, which includes 3 and 10% acrylamide concentrations used to control the enzyme purity [49]. All experiments of the CA inhibition assay were repeated three times and the results are given as the arithmetic mean of these repetitions.

## 3. Results

### 3.1. Chemistry

The synthesis of compounds **17**–**24** was obtained from the reaction of carbonyl compounds with primary amines, benzophenone hydrazone, or phenylhydrazine. For this, Schiff bases were synthesized from the reaction of isovanillin (**1**) with *N*^1^,*N*^1^-dimethylbenzene-1,4-diamine (**2**), 2-aminophenol (**3**), 4-fluoroaniline (**4**), 4-bromoaniline (**5**), p-phenylenediamine (**6**), benzlyamine (**7**), benzophenone hydrazone (**8**), or phenylhydrazine (**9**) (1 mmol). (1 mmol) at a ratio of 1:1. Reactions were recorded with TLC. The percentage yield of synthetic Schiff bases (**10**–**15**) and hydrazineylidene derivatives (**16** and **17**) was excellent. The IR spectra of the synthesized Schiff bases were conducted in the range 500–4000 cm^−1^. The existence of stretched C=N bands at 1600–1660 cm^−1^ [50] and the nonexistence of carbonyl (C=O) at 1700 cm^−1^ were confirmed by the infrared spectra of the synthesized compounds, whereas NH is cleared away or hidden underneath the broad bands at 3450–3300 cm^−1^ in Schiff bases. Proton of Schiff bases’ gives a singlet in the region 8.59–7.60 ppm in the ^1^H NMR spectrum. The protons of the hydroxyl group (-OH) in the compounds **12**, **13,** and **16** were observed at 6.14 (**12**), 5.72 (**13**), and 5.79 (**16**) ppm, respectively. Additionally, aromatic protons were resonated in the region between 7.79–6.71 ppm. In the ^13^C NMR spectra of the new Schiff bases, the carbon atoms (HC=N) were recorded between 147.15 and 166.11 ppm, another result supporting the fact that the proposed structures were obtained from elemental analysis, and the results are in agreement with the formulas of the proposed Schiff bases.

### 3.2. Antioxidant Results

The excessive formation of free radicals and ROS in living organisms disrupts the structure of many cellular biomolecules and may cause some degenerative diseases [51]. Therefore, oxidative stress and ROS pose significant risks for many chronic diseases such as cancer, cardiovascular diseases, immunodeficiency syndrome, age-related pathologies, arteriosclerosis, diabetes mellitus, and obesity [52]. Antioxidants eliminate undesirable harmful effects of ROS and free radicals even at low concentrations. Additionally, they reduce oxidative stresses in the human body or food systems and prevent harmful effects of ROS [53].

There are many methods for the determination of the antioxidant effectiveness of newly synthesized molecules. In this study, we selected putative and prominent methods including ABTS^•+^ and DPPH^•^ scavenging activity, and Fe^3+^, Fe^2+^-TPTZ complex, and Cu^2+^reducing abilities. The reduction potentials of Schiff bases (**10**–**15**) and hydrazineylidene derivatives (**16** and **17**) were realized with three different reduction assays. In this study, Fe^3+^ reducing by compounds (**10**–**17**) led to the formation of an Fe_4_[Fe(CN)_6_] complex, which demonstrated absorbance at 700 nm [53]. In this way, Schiff bases (**10**–**15**) and hydrazineylidene derivatives (**16** and **17**) can have reducing capabilities, and neutralize free radicals and ROS. As shown in Table 1 and Supplementary Figure S1A, Schiff bases (**10**–**15**) and hydrazineylidene derivatives (**16** and **17**) exhibited a potent Fe^3+^ reducing ability. However, the Fe^3+^ reducing effect of 30 μg/mL Schiff bases (**10**–**15**) and hydrazineylidene derivatives (**16** and **17**) and standards were ordered as **10** (2.827 ± 0.016, r^2^: 0.9912) > **14** (2.741 ± 0.026, r^2^: 0.9989) > **11** (2.591 ± 0.010, r^2^: 0.9822) > BHA (2.448 ± 0.021, r^2^: 0.9984) > **17** (2.391 ± 0.037, r^2^: 0.9959) > BHT (1.994 ± 0.033, r^2^: 0.9932) > Trolox (1.570 ± 0.016, r^2^: 0.9915) > α-Tocopherol (1.446 ± 0.009, r^2^: 0.9665) > **15** (0.363 ± 0.021, r^2^: 0.9748) > **16** (0.311 ± 0.018, r^2^: 0.9839) > **12** (0.294 ± 0.008, r^2^: 0.9742) > **8** (0.244 ± 0.005, r^2^: 0.9632) > **13** (0.198 ± 0.004, r^2^: 0.9611). The results show that all Schiff bases (**10**–**15**) and hydrazineylidene derivatives (**16** and **17**) possessed marked reductive potentials. Schiff bases **10**, **14, and 11** and hydrazineylidene **17** demonstrated higher activity when compared with α-tocopherol and Trolox. In particular, (*E*)-5-(((4-(dimethylamino)phenyl)imino)methyl)-2-methoxyphenol (**10**) exhibited powerful reducing power (2.827 ± 0.016, r^2^: 0.9912). Additionally, this activity was found to be higher than BHA (2.448 ± 0.021, r^2^: 0.9984) and BHT (1.994 ± 0.033, r^2^: 0.9932). However, the lowest reducing activity was observed in Schiff base **13,** which contained a bromo group. It is known that phenols and their metabolites have many biological activities including free radical scavenging, singlet oxygen quenching, metal binding, and reducing power properties [54].

The Cu^2+^ reducing abilities of Schiff bases (**10**–**15**) and hydrazineylidene derivatives (**16** and **17**) are summarized in Table 1 and Supplementary Figure S1B. A good correlation was observed between the Cu^2+^ reducing ability and Schiff base (**10**–**15**) and hydrazineylidene derivative (**16** and **17**) concentrations. At 30 μg/mL, the absorbance values of reducing ability exhibited by Schiff bases (**10**–**15**) and hydrazineylidene derivatives (**16** and **17**) were found as following order: **11** (2.332 ± 0.018, r^2^: 0.9997) > BHA (2.268 ± 0.011, r^2^: 0.9956 > **10** (2.176 ± 0.044, r^2^: 0.9931) > BHT (2.149 ± 0.019, r^2^: 0.9971) > **17** (2.026 ± 0.016, r^2^: 0.9610) > **14** (1.970 ± 0.018 ± 0.056, r^2^: 0.9876) > α-Tocopherol (1.923 ± 0.032, r^2^: 0.9972) > **16** (1.266 ± 0.029, r^2^: 0.9932) > Trolox (1.174 ± 0.027, r^2^: 0.9738) > **8** (1.005 ± 0.009, r^2^: 0.9673) > **12** (0.951 ± 0.010, r^2^: 0.9795) > **13** (0.875 ± 0.020, r^2^: 0.9833) > **15** (0.803 ± 0.013, r^2^: 0.9763). As in Fe^3+^ reduction, in this reduction method, the highest reducing activity was exhibited by **11,** and the lowest reducing activity was shown by **15**. The presence of -OH groups in phenolic rings increases the reducing activity properties of compounds [1].

Aside from Fe^3+^ and Cu^2+^ reduction properties, the Fe^3+^-TPTZ reducing ability of Schiff bases (**10**–**15**) and hydrazineylidene derivatives (**16** and **17**) was studied, and is summarized in Table 1 and Supplementary Figure S1C. Additionally, a positive correlation was displayed between the reducing abilities and used concentrations (10–30 μg/mL). At 30 μg/mL, the Fe^3+^-TPTZ ability of Schiff bases (**10**–**15**) and hydrazineylidene derivatives (**16** and **17**) and standards declined in the following order (Table 1 and Supplementary Figure S1B): **11** (2.183 ± 0.016, r^2^: 0.9921) > BHA (2.156 ± 0.005, r^2^: 0.9565) > **17** (2.121 ± 0.014, r^2^: 0.9815) > **10** (2.076 ± 0.004, r^2^: 0.9897) ≈ **14** (2.074 ± 0.017, r^2^: 0.9917) > Trolox (2.051 ± 0.028, r^2^: 0.9931) > BHT (2.037 ± 0.027, r^2^: 0.9782) > α-Tocopherol (1.763 ± 0.026, r^2^: 0.9828 > **16** (1.069 ± 0.057, r^2^: 0.9897) > **12** (0.806 ± 0.003, r^2^: 0.9569) > **8** (0.728 ± 0.020, r^2^: 0.9561) > **13** (0.701 ± 0.003, r^2^: 0.9648) > **15** (0.646 ± 0.034, r^2^: 0.9523). Like other reduction tests, this is low-cost, rapid, stable, and selective for pure compounds, regardless of hydrophobicity and chemical ingredient.

**Scheme 2:**
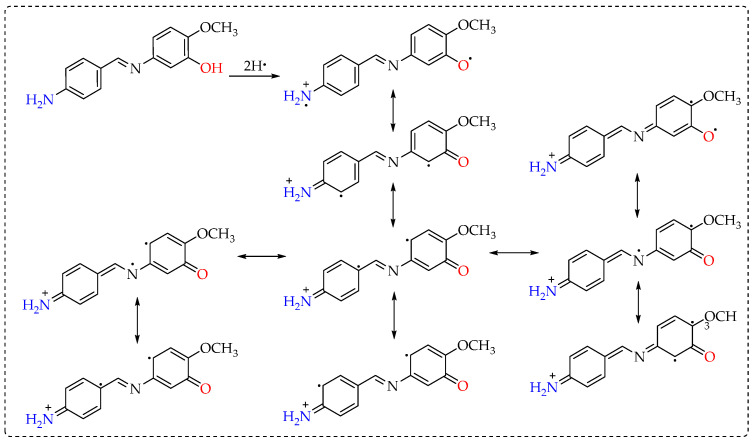
Proposed radical removing mechanism of molecule **14**.

There are many radical scavenging ability methods for the evaluation of the antioxidant power of pure and newly synthesized compounds. In this study, we selected two putative and common methods including DPPH‧ scavenging and ABTS^•+^ scavenging activities. Additionally, as given in Table 2 and Supplementary Figure S2A, Schiff bases (**10**–**15**) and hydrazineylidene derivatives (**16** and **17**) demonstrated statistically significant effective DPPH radical ability (*p* < 0.01). The DPPH radical activity of Schiff bases (**10**–**15**) and hydrazineylidene derivatives (**16** and **17**) and positive controls increased depending on increased Schiff base (**10**–**15**) and hydrazineylidene derivative (**16** and **17**) concentrations. The half maximal scavenging concentration (IC_50_) of Schiff bases (**10**–**15**) and hydrazineylidene derivatives (**16** and **17**) and standards toward DPPH radicals increased in the following order: **15** (IC_50_: 99.010 μg/mL; r^2^: 0.9980) < **13** (IC_50_: 87.721 μg/mL; r^2^: 0.9922) < **8** (IC_50_: 86.625 μg/mL; r^2^: 0.9989) < **12** (IC_50_: 57.750 μg/mL; r^2^: 0.9998) < **16** (IC_50_: 30.130 μg/mL; r^2^: 0.9723) < **10** (IC_50_: 16.902 μg/mL; r^2^: 0.9687 < **17** (IC_50_: 14.744 μg/mL; r^2^: 0.9874) < **14** (IC_50_: 13.860 μg/mL; r^2^: 0.9629) < BHT (IC_50_: 13.326 μg/mL; r^2^: 0.9734) < Trolox (IC_50_: 12.157 μg/mL; r^2^: 0.9645) ≈ **11** (IC_50_: 12.157 μg/mL; r^2^: 0.9636) < BHA (IC_50_: 11.550 μg/mL; r^2^: 0.9690) < α-Tocopherol (IC_50_: 10.043 μg/mL; r^2^: 0.9760). The results demonstrated that the DPPH radical activity of Schiff bases (**10**–**15**) and hydrazineylidene derivatives (**16** and **17**) was close to the standards. However, the most powerful DPPH radical scavenging value was calculated for **11** (IC_50_: 12.157 μg/mL; r^2^: 0.9636), which is similar to Trolox (IC_50_: 12.157 μg/mL; r^2^: 0.9645) and lower than BHA (IC_50_: 11.550 μg/mL; r^2^: 0.9690) and α-Tocopherol (IC_50_: 10.043 μg/mL; r^2^: 0.9760). As an example of the radical scavenging mechanism for Schiff bases (**10**–**15**) and hydrazineylidene derivatives (**16** and **17**), the proposed radical scavenging mechanism of molecule **14** is given in Scheme 2.

The ABTS^•+^ scavenging assay was used for the radical removing ability of the compounds. As shown in Supplementary Figure S2B and Table 1, the results demonstrate that Schiff bases (**10**–**15**) and hydrazineylidene derivatives (**16** and **17**) had higher ABTS^•+^ scavenging ability as follows: **13** (IC_50_: 34.650 μg/mL; r^2^: 0.9901) < **15** (IC_50_: 23.896 μg/mL; r^2^: 0.9979) < **12** (IC_50_: 27.720 μg/mL; r^2^: 0.9982) < **10** (IC_50_: 14.437 μg/mL; r^2^: 0.9767 < **8** (IC_50_: 12.157 μg/mL; r^2^: 0.9549) < **16** (IC_50_: 11.745 μg/mL; r^2^: 0.9838) < α-Tocopherol (IC_50_: 9.493 μg/mL; r^2^: 0.9889) < **14** (IC_50_: 5.330 μg/mL; r^2^: 0.9498) < BHT (IC_50_: 4.950 μg/mL; r^2^: 0.9633) < Trolox (IC_50_: 4.846 μg/mL; r^2^: 0.9769) < BHA (IC_50_: 4.470 μg/mL; r^2^: 0.9702) < **11** (IC_50_: 4.386 μg/mL; r^2^: 0.9701) < **17** (IC_50_: 4.304 μg/mL; r^2^: 0.9711). The low IC_50_ values reflect effective ABTS^•+^ scavenging ability [55].

All the synthesized Schiff bases (**10**–**15**) and hydrazineylidene derivatives (**16** and **17**) displayed in vitro inhibition effects against cytosolic hCA I, which is associated with cerebral and retinal edema, hCA II, which is associated with edema, glaucoma, epilepsy, and mountain sickness, and AChE and BChE, which have been linked with AD for their inhibition efficacy. Mountain sickness is a disease that affects mountaineers or travelers at high altitudes who do not have enough time to acclimatize to altitudes above 2400 m [56,57]. They often develop symptoms including headaches, appetite loss, nausea, poor sleep, gastrointestinal distress, and general malaise due to low oxygen levels. In some cases, mountain sickness can cause brain edema and even death. The CA inhibitory effects of the Schiff bases (**10**–**15**) and hydrazineylidene derivatives (**16** and **17**) were determined using an esterase assay [53] and compared to acetazolamide (AZA). For the determination of the synthesized Schiff bases’ (**10**–**15**) and hydrazineylidene derivatives’ (**16** and **17**) action towards AChE and BChE, Ellman’s procedure [45] was employed, and compared to the standard inhibitor of Tacrine. Further, the following insights can be gleaned from the studied enzyme inhibition results given in Table 3 and Table 4.

The synthesized Schiff bases (**10**–**15**) and hydrazineylidene derivatives (**16** and **17**) exhibited effective inhibition profiles against widespread cytosolic hCA I isozyme with Ki values ranging from 26.08 ± 8.53 nM to 85.79 ± 24.80 nM. Isovanilin (**1**), as our starting material, showed a lesser inhibition profile (Ki: 112.30 ± 25.75 nM) when compared to the synthesized Schiff bases (**10**–**15**) and hydrazineylidene derivatives (**16** and **17**). However, within this series, the compounds (E/Z)-5-(((4-aminophenyl)imino)methyl)-2-methoxyphenol (**14**) were found to be the best inhibitor (Ki: 26.08 ± 8.53 nM) towards cytosolic hCA I isozyme in comparison with AZA (K_i_: 35.39 ± 9.01 nM). However, the compounds **17, 13,** and **14** showed stronger inhibition ability than AZA. Common to all hCA isomers of the α-CA family is a highly conserved active site, which a Zn^2+^ ion coordinated by His94, His96, and His119 residues and an H_2_O molecule. Most hCA inhibitors have been identified as Zn^2+^-binding molecules. Overexpression of the hCA I isozyme has been associated with cerebral and retinal edema, while the hCA II isoform has been associated with altitude sickness, glaucoma, and epilepsy [58].

All synthesized Schiff bases (**10**–**15**) and hydrazineylidene derivatives (**16** and **17**) inhibited cytosolic and dominant hCA II isozyme with K_i_s of 67.59 ± 15.70–164.72 ± 29.46 nM. Among them, the compound of (*E*)-5-(((4-bromophenyl)imino)methyl)-2-methoxyphenol (**13**) showed important inhibition effects towards hCA II with a Ki of 67.59 ± 15.70 nM. However, the inhibition profiles of all synthesized Schiff bases (**10**–**15**) and hydrazineylidene derivatives (**16** and **17**) were found to be lower than AZA (Ki: 16.60 ± 1.29 nM). Additionally, the selectivity index (hCA I/hCA II) for both hCA isoenzymes shows that the synthesized substances have a higher affinity for the hCA I isozyme than hCA II isoforms. However, the rest of the Schiff bases demonstrated moderate inhibition of ubiquitous and dominant cytosolic hCA II isoenzyme.

The AChE inhibition abilities of Schiff bases (**10**–**15**) and hydrazineylidene derivatives (**16** and **17**) were given for the first time in this study. The results are presented and summarized in Table 4. Tacrine was used as positive control for AChE inhibition with a Ki of 68.6 ± 3.8 nM towards AChE enzyme. As presented in Table 4, IC_50_ values of all the Schiff bases (**17**–**24**) were in the range of 16.11 to 57.75 nM towards AChE. The inhibition results showed by most of synthesized Schiff bases (**10**–**15**) and hydrazineylidene derivatives (**16** and **17**) had higher inhibitory effects than the TAC (IC_50_: 46.20 nM).

Additionally, BChE was highly inhibited by the Schiff bases (**10**–**15**) and hydrazineylidene derivatives (**16** and **17**), with IC_50_s in the range of 19.80 to 53.31 nM (Table 4). These results clearly show that the synthesized Schiff bases (**10**–**15**) and hydrazineylidene derivatives (**16** and **17**) displayed effective BChE inhibition abilities. However, the most powerful BChE inhibition was observed for (*E*)-2-methoxy-5-((2-phenylhydrazineylidene)methyl)phenol (**17**) with an IC_50_ value of 19.80 nM. For comparison, Tacrine, the first centrally acting BChE inhibitor, exhibited an IC_50_ of 24. 75 nM (r^2^: 0.9878) against BChE enzyme. Tacrine has been removed from the market due to some undesirable side effects, especially hepatotoxicity, in a significant number of patients [58].

## 4. Discussion

In this study, the synthesis of Schiff bases using the microwave method, and their antioxidant capacity and some metabolic enzyme inhibition properties were reported for the first time. Here, six Schiff bases (**10**–**15**) and two hydrazineylidene derivatives (**16** and **17**) were synthesized using an environmentally friendly methodology. A simple, efficient, and fast method was applied for synthesis that does not include solvents or catalysts. Compared to other methods, microwave irradiation is the simplest and cheapest way to synthesize novel Schiff bases.

Schiff bases are an important class of organic compounds that are commonly used and researched due to their unique structural properties and biological activities. Additionally, they have numerous applications, especially in biochemistry and medicine [59]. It is known that Schiff bases have received great attention due to their many biological and pharmaceutical activities [60] such as antifungal, antiviral, antitumor, antibacterial, antimalarial, anticancer, and anti-inflammatory activities and enzyme inhibition properties [61,62,63,64]. The biological activity of Schiff bases originates from the imine or azomethine (-C=N-) functional groups, as well as hydrophobic aromatic groups, and they can coordinate easily with metals to form versatile functional complexes [65]. The enzyme inhibition properties of Schiff bases were tested against very important metabolic enzymes such as CA, AChE, and BChE, which are associated with some global disorders, and it was observed that they effectively inhibited them. Recently, three series of symmetrical Schiff bases and their amine derivatives were tested towards AChE and hCA I and II isoenzymes, and demonstrated nanomolar inhibition profiles against the indicated metabolic enzymes, which have a significant role in drug discovery and design as well as in toxicology and medicine [59]. It was reported that Schiff bases, as kind of compounds containing azomethine groups, exhibited antioxidant activity, especially O_2_^‧–^ scavenging activity. In this context, it has been observed that Schiff base complexes containing copper can almost completely remove the existing O_2_^‧–^ even at low concentrations [66].

In another study, the antioxidant activity of some resveratrol analogues including 4′-hydroxyphenyl-benzo[d]thiazole, p-(*N,N*-dimethyl)aminobenzylidene-2-aminothiophenol, and p-nitrobenzylidene-2-aminothiophenol were synthesized, characterized, and their antioxidant activity was evaluated [67]. Better antioxidant and antifungal activity of chitosan derivatives bearing Schiff bases were reported. In a recent study, many chitosan derivatives containing Schiff bases were synthesized. In this study, the structural characterization of chloracetyl chitosan oligosaccharide derivatives grafted with pyridine-4-aldehyde Schiff bases was performed, and their antioxidant activities against DPPH‧ radical and O_2_^‧–^ were determined [68]. Similarly, it was indicated that some chiral selenite ligands and their palladium complexes have antioxidant activity, which increased with concentration [69]. In a recent study, it was shown that the Co^2+^ and Fe^2+^ complexes of Schiff bases demonstrated effective antioxidant ability using different methods including the FRAP and CUPRAC reducing methods, and ABTS and DPPH radical scavenging methods [60]. Similarly, some Schiff base ligands ((*E*)-6-methyl-2-(2,3,4-trimethoxybenzylideneamino)-4,5,6,7-tetrahydrobenzo[b]-thiophene-3-carbonitrile) and their Co^2+^ and Pd^2+^ complexes exhibited powerful antioxidant abilities [10]. The transition metal complexes from bidentate Schiff base ligands containing both amine (–NH_2_) and –OH groups have been extensively studied. In particular, Schiff base–metal complexes containing nitrogen and oxygen donor atoms show many application areas such as catalytic and biological activities [10]. Antioxidants containing such Schiff bases significantly prevent the formation and accumulation of free radicals, and protect the body from oxidative damage. In a recent study, Schiff bases and new secondary amine derivatives of p-vanillin demonstrated powerful antioxidant abilities by strongly scavenging ABTS (IC_50_: 1.25–464.38 mM) and DPPH radicals (IC_50_: 2.20–870.78 mM) and by exhibiting strong Fe[(CN)_6_]^3+^ reducing ability [70].

When the relevant literature is examined, it can be observed that Schiff bases inhibit some metabolic enzymes that are seriously important. This situation holds great promise in the design and synthesis of new drugs for some diseases that are very common in the global context. In studies carried out in this context, symmetrical Schiff bases were synthesized from 1,2-diaminoethane, 1,3-diaminopropane, and 1,4-diaminobutane and substituted benzaldehydes effectively inhibited the cytosolic CA I and II isozymes and AChE, with Ki values in the range of 159.43 ± 30.03 to 563.73 ± 115.30 nM for hCA I, 104.88 ± 18.44 to 524.32 ± 95.03 nM for hCA II, and 3.95 ± 0.74 to 30.83 ± 6.81 nM for AChE [71]. Similarly, Co^2+^ and Fe^2+^ complexes of Schiff bases demonstrated Ki values in the range of 1.06 ± 0.16 to 9.83 ± 0.74 nM for hCA I, 0.68 ± 0.12 to 7.16 ± 1.14 nM for hCA II, 44.66 ± 10.06 to 78.34 ± 17.83 nM for AChE,50.36 ± 13.88 to 88.36 ± 20.03 nM for BChE, and 33.72 ± 7.93 to 90.56 ± 27.52 nM for α-glycosidase enzyme [56]. Some Schiff base ligands ((*E*)-6-methyl-2-(2,3,4-trimethoxybenzylideneamino)-4,5,6,7-tetrahydrobenzo[b]-thiophene-3-carbonitrile) and their Co^2+^ and Pd^2+^ complexes exhibited IC_50_ values in the range of 98.86–153.25 μM against glutathione S-transferase (GST), 50.47–88.22 μM towards AChE, and 88.76–120.72 μM on BChE [10].

In conclusion, the newly synthesized Schiff bases are promising potential antioxidant agent candidates for the scavenging of ROS, which cause damage in humans. Additionally, we believe that these results may be useful for the synthesis of new hCA I and II isoenzymes, AChE and BChE inhibitors, and in the development of drugs for the treatment of some common and global diseases including edema, epilepsy, glaucoma, mountain sickness, and AD.

## Figures and Tables

**Table 1:** The reducing ability of 30 µg/mL of Schiff bases (**10**–**15**) and hydrazineylidene derivatives (**16** and **17**) through Fe^3+^-TPTZ, Fe^3+^, and Cu^2+^ reducing methods.

Antioxidants	Fe^3+^ Reducing		Cu^2+^ Reducing		Fe^3+^-TPTZ Reducing	
	λ_700_ *	r^2^	λ_450_ *	r^2^	λ_593_ *	r^2^
BHA	2.448 ± 0.021	0.9984	2.268 ± 0.011	0.9956	2.156 ± 0.005	0.9565
BHT	1.994 ± 0.033	0.9932	2.149 ± 0.019	0.9971	2.037 ± 0.027	0.9782
Trolox	1.570 ± 0.016	0.9915	1.174 ± 0.027	0.9738	2.051 ± 0.028	0.9931
α-Tocopherol	1.446 ± 0.009	0.9665	1.923 ± 0.032	0.9972	1.763 ± 0.026	0.9828
**1**	0.244 ± 0.005	0.9632	1.005 ± 0.009	0.9673	0.728 ± 0.020	0.9561
**10**	2.827 ± 0.016	0.9912	2.176 ± 0.044	0.9931	2.076 ± 0.004	0.9897
**11**	2.591 ± 0.010	0.9822	2.332 ± 0.018	0.9997	2.183 ± 0.016	0.9921
**12**	0.294 ± 0.008	0.9742	0.951 ± 0.010	0.9795	0.806 ± 0.003	0.9569
**13**	0.198 ± 0.004	0.9611	0.875 ± 0.020	0.9833	0.701 ± 0.003	0.9648
**14**	2.741 ± 0.026	0.9989	1.970 ± 0.018	0.9876	2.074 ± 0.017	0.9917
**15**	0.363 ± 0.021	0.9748	0.803 ± 0.013	0.9763	0.646 ± 0.034	0.9523
**16**	0.311 ± 0.018	0.9839	1.266 ± 0.029	0.9932	1.069 ± 0.057	0.9897
**17**	2.391 ± 0.037	0.9959	2.026 ± 0.016	0.9610	2.121 ± 0.014	0.9815

* expressed as absorbance values.

**Table 2:** IC_50_ (μg/mL) values for DPPH^•^ and ABTS^•+^ scavenging abilities of some Schiff bases (**10**–**15**), hydrazineylidene derivatives (**16** and **17**), and standards.

Antioxidants	DPPH^•^ Scavenging		ABTS^•+^ Scavenging	
	IC_50_	r^2^	IC_50_	r^2^
BHA	11.55	0.9690	4.47	0.9702
BHT	13.32	0.9734	4.95	0.9633
Trolox	12.15	0.9645	4.84	0.9769
α-Tocopherol	10.04	0.9760	9.49	0.9889
**1**	86.62	0.9989	12.15	0.9549
**10**	16.90	0.9687	14.43	0.9767
**11**	12.15	0.9636	4.38	0.9701
**12**	57.75	0.9998	27.72	0.9982
**13**	87.72	0.9922	34.65	0.9901
**14**	13.86	0.9629	5.33	0.9498
**15**	99.01	0.9980	23.89	0.9979
**16**	30.13	0.9723	11.74	0.9838
**17**	14.74	0.9874	4.30	0.9711

**Table 3:** The summarized inhibition parameters of synthesized Schiff bases (**10**–**15**) and hydrazineylidene derivatives (**16** and **17**) towards human carbonic anhydrase I and II isoforms (hCA I and II).

Compounds	IC_50_ (nM)				Selectivity Index (hCA I/hCA II)
	hCA I	r^2^	hCA II	r^2^	
**1**	138.60	0.9820	173.25	0.9770	0.340
**10**	66.00	0.9965	130.75	0.9691	0.372
**11**	86.62	0.9810	88.22	0.9766	0.730
**12**	61.87	0.9823	99.00	0.9794	0.424
**13**	63.00	0.9872	77.00	0.9930	0.483
**14**	53.30	0.9801	69.30	0.9883	0.306
**15**	99.00	0.9840	138.60	0.9732	0.521
**16**	69.30	0.9839	111.77	0.9738	0.326
**17**	96.25	0.9933	115.50	0.9689	0.247
Acetazolamide	46.20	0.9952	24.75	0.9878	1.167

**Table 4:** The inhibition parameters of Schiff bases (**10**–**15**) and hydrazineylidene derivatives (**16** and **17**) towards AChE and BChE.

Compounds	IC_50_ (nM)				Selectivity Index (AChE/BChE)
	AChE	r^2^	BChE	r^2^	
**1**	57.75	0.9823	53.31	0.9946	1.083
**10**	26.65	0.9947	23.89	26.653	1.115
**11**	22.35	0.9911	34.65	22.354	0.645
**12**	23.10	0.9868	33.00	23.100	0.700
**13**	17.76	0.9720	28.87	17.769	0.615
**14**	34.65	0.9980	38.50	34.650	0.900
**15**	25.66	0.9991	36.47	25.666	0.704
**16**	16.11	0.9801	25.66	16.116	0.627
**17**	23.89	0.9986	19.80	23.896	1.206
Tacrine	46.20	0.9952	24.75	0.9878	1.664

## Data Availability

Data are provided in a publicly accessible repository.

## Supplementary Materials

The following supporting information can be downloaded at: https://www.mdpi.com/article/10.3390/pharmaceutics15030779/s1, ^1^H NMR and ^13^C NMR spectra of synthesized compounds. Figure S1: The reducing ability of 10–30 µg/mL of Schiff bases (**10**–**15**), hydrazineylidene derivatives (**16**–**17**) and standards. (A) Fe^3+^ reducing effect, (B) Cu^2+^ reducing effect, (C) Fe^3+^-TPTZ reducing effect. Figure S2: Radical removing abilities of different concentrations (10–30 µg/mL) of Schiff bases (**10**–**15**), hydrazineylidene derivatives (**16**–**17**), and standards. (**A**) DPPH‧ removing effect, (**B**) ABTS•+ removing effect.
