# Supplementary material for: Synthesis of Schiff Bases Containing Phenol Rings and Investigation of Their Antioxidant Capacity, Anticholinesterase, Butyrylcholinesterase, and Carbonic Anhydrase Inhibition Properties
Source: Pharmaceutics. 2023 Feb 26;15(3):779. doi: 10.3390/pharmaceutics15030779 (PMC10051454; doi:10.3390/pharmaceutics15030779)
Supplement: Supplementary file 1 [file pharmaceutics-15-00779-s001.zip › pharmaceutics-2184132-supplementary.pdf]

NMR SPECTRUM AND MASS

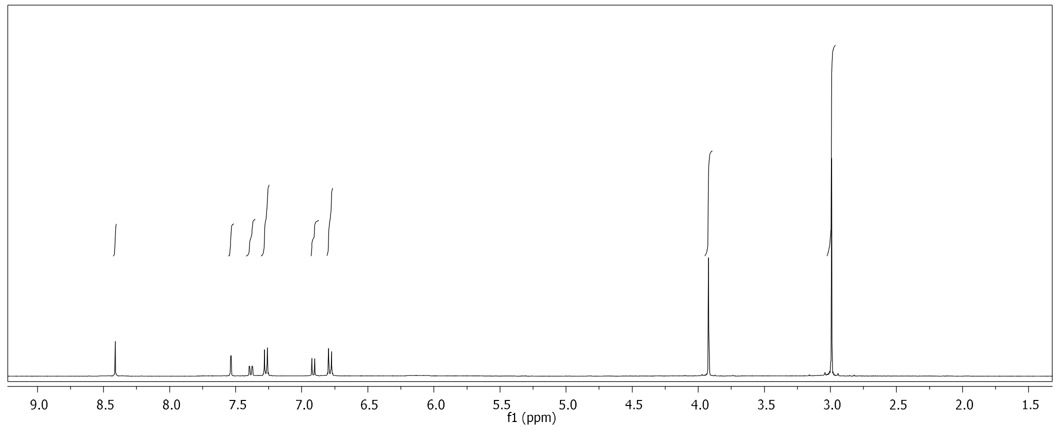

^{1}H NMR of (*E*)-5-(((4-(dimethylamino)phenyl)imino)methyl)-2-methoxyphenol **(10)** in $CDCl_3$

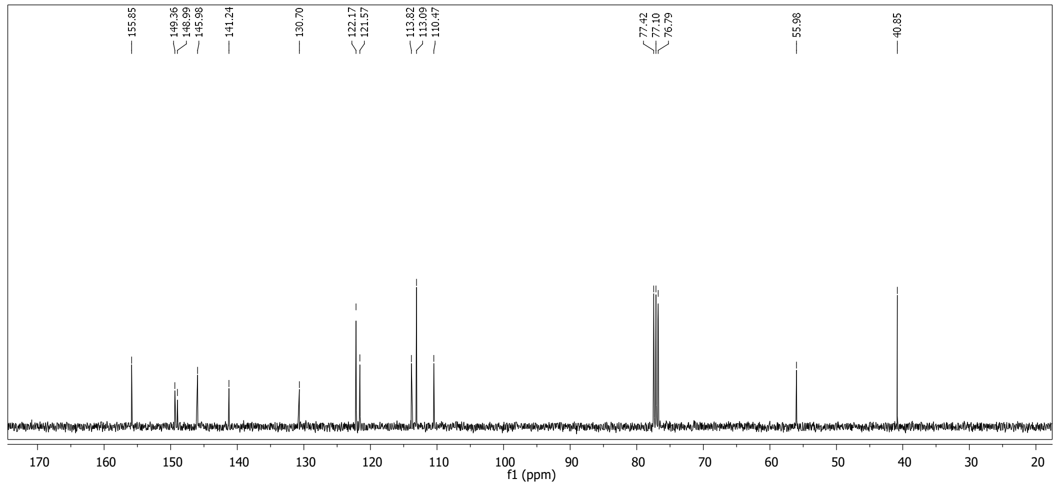

^{13}C NMR of (*E*)-5-(((4-(dimethylamino)phenyl)imino)methyl)-2-methoxyphenol **(10)** in $CDCl_3$

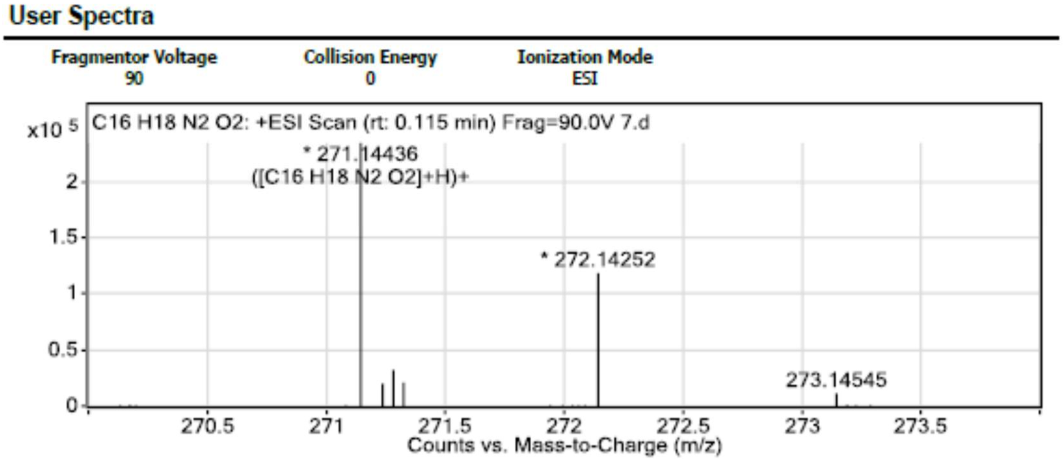

Mass spektrum of (*E*)-5-(((4-(dimethylamino)phenyl)imino)methyl)-2-methoxyphenol **(10)**

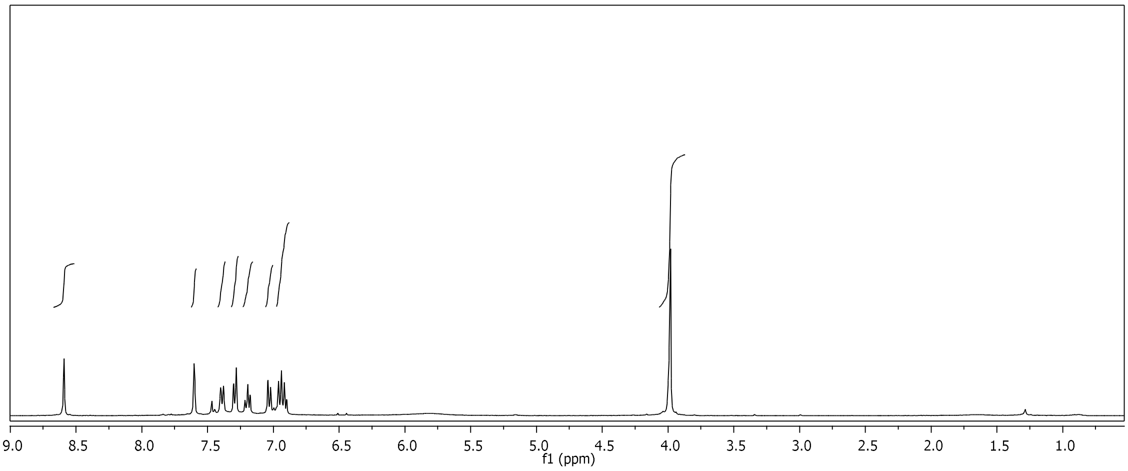

^{1}H NMR of (*E*)-5-(((2-hydroxyphenyl)imino)methyl)-2-methoxyphenol **(11)** in $CDCl_3$

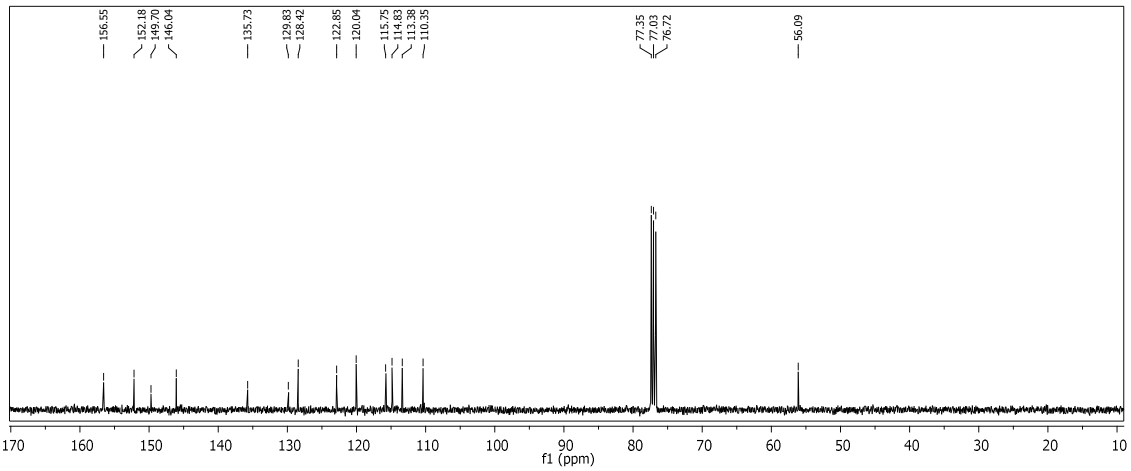

^{13}C NMR of (*E*)-5-(((2-hydroxyphenyl)imino)methyl)-2-methoxyphenol **(11)** in $CDCl_3$

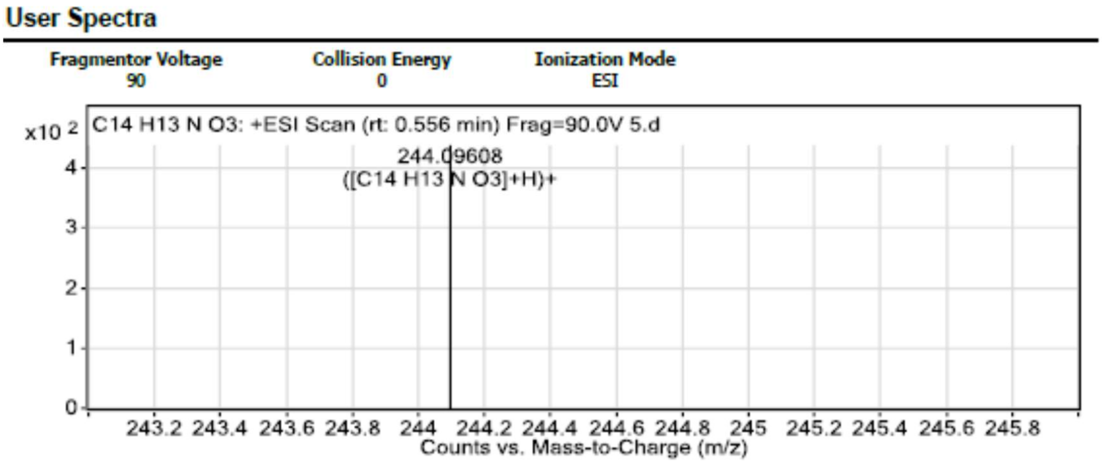

Mass spektrum of (E)-5-(((2-hydroxyphenyl)imino)methyl)-2-methoxyphenol **(11)**

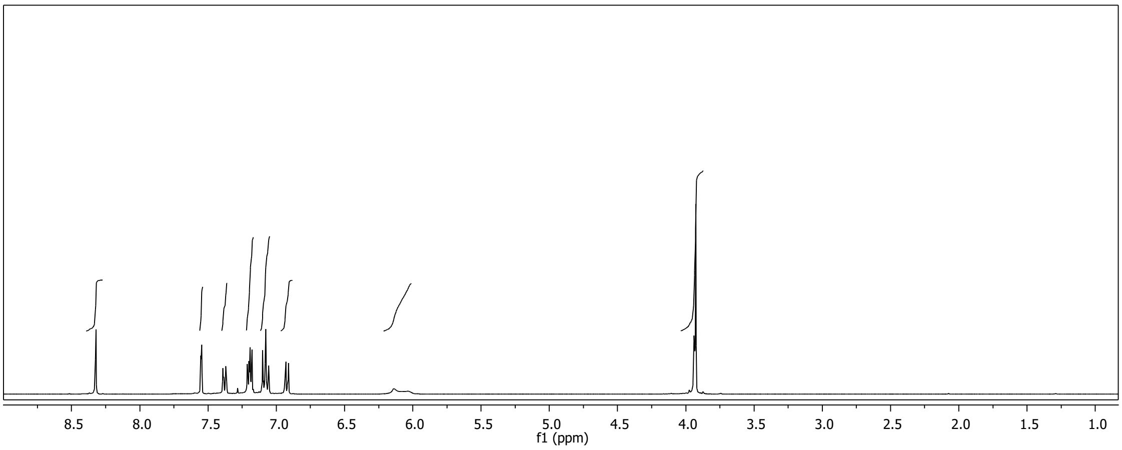

^{1}H NMR of (*E*)-5-(((4-fluorophenyl)imino)methyl)-2-methoxyphenol **(12)** in $CDCl_3$

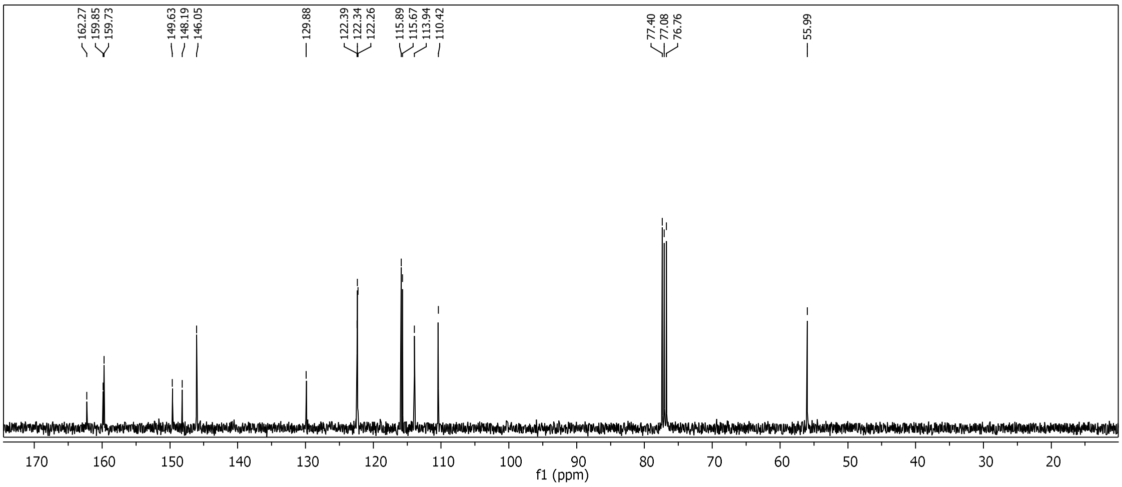

^{13}C NMR of (*E*)-5-(((4-fluorophenyl)imino)methyl)-2-methoxyphenol **(12)** in $CDCl_3$

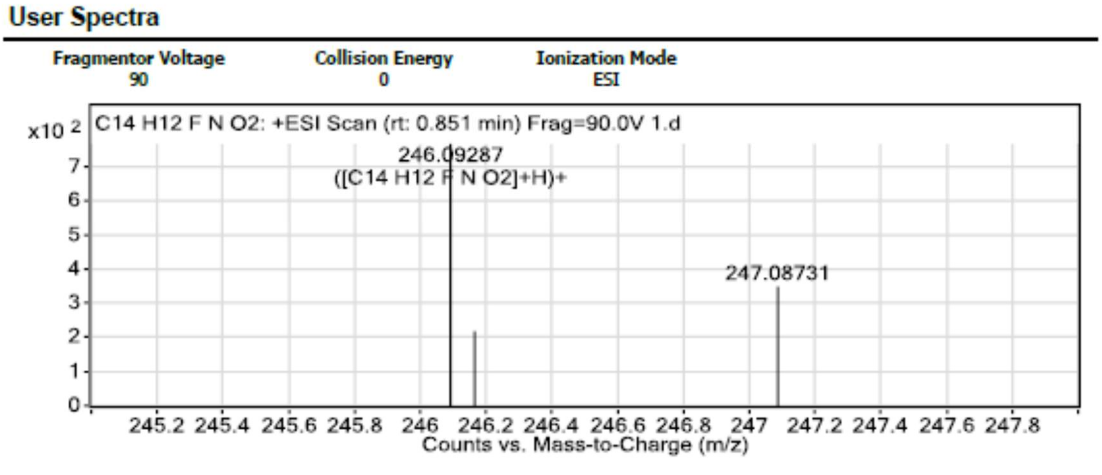

Mass spektrum of (*E*)-5-(((4-fluorophenyl)imino)methyl)-2-methoxyphenol **(12)**

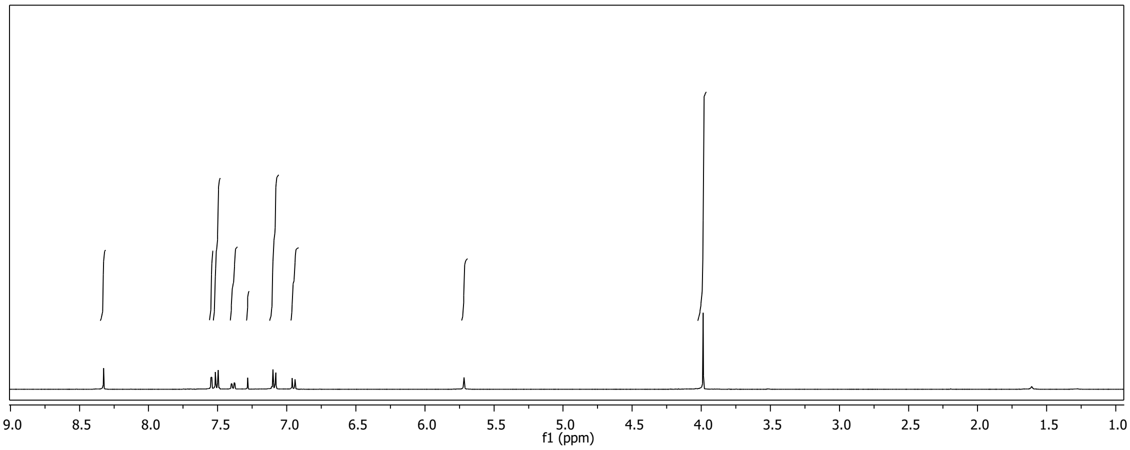

^1H NMR of (E)-5-(((4-bromophenyl)imino)methyl)-2-methoxyphenol **(13)** in $CDCl_3$

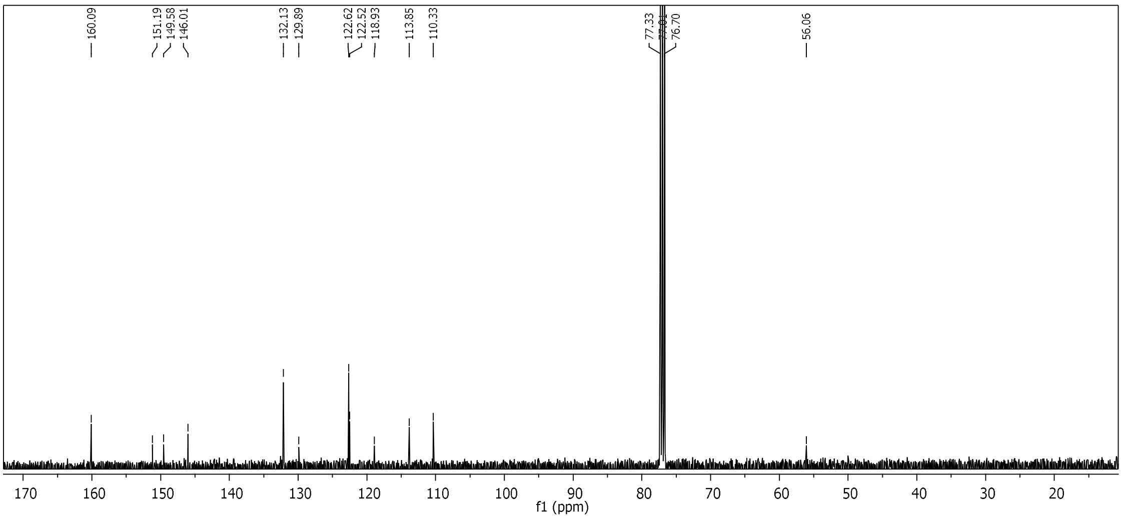

^{13}C NMR of (*E*)-5-(((4-bromophenyl)imino)methyl)-2-methoxyphenol **(13)** in $CDCl_3$

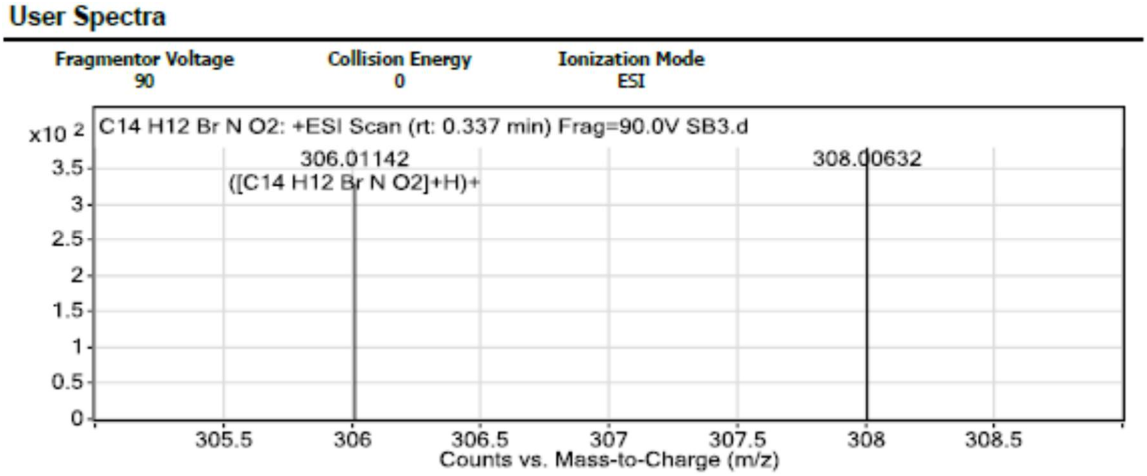

Mass spektrum of (*E*)-5-(((4-bromophenyl)imino)methyl)-2-methoxyphenol **(13)**

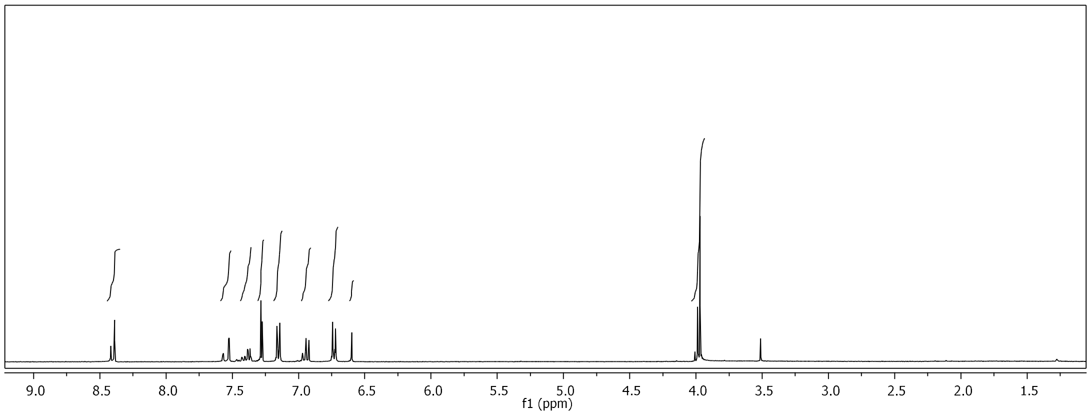

^{1}H NMR of (*E/Z*)-5-(((4-aminophenyl)imino)methyl)-2-methoxyphenol **(14)** in $CDCl_3$

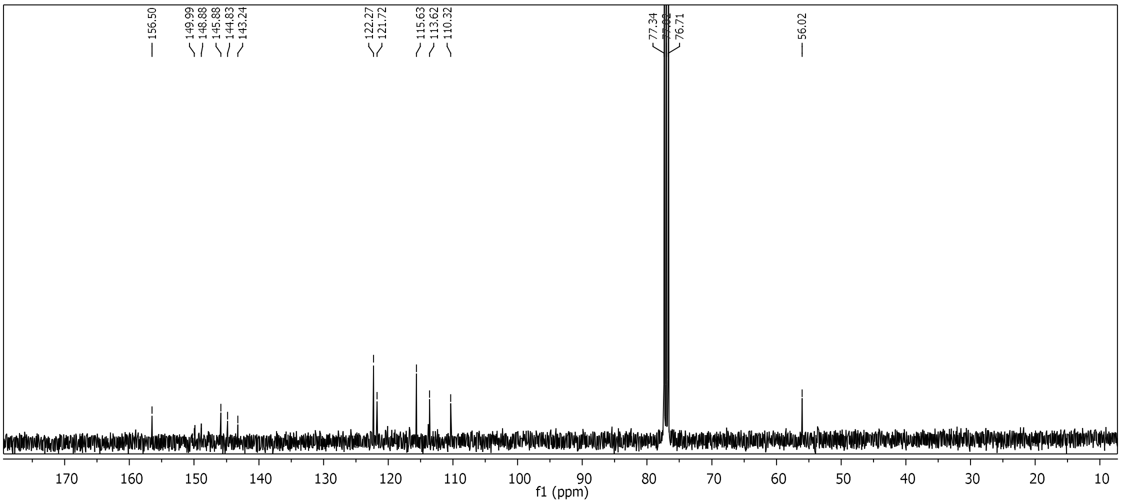

^{13}C NMR of **(*E/Z*)**-5-(((4-aminophenyl)imino)methyl)-2-methoxyphenol **(14)** in $CDCl_3$

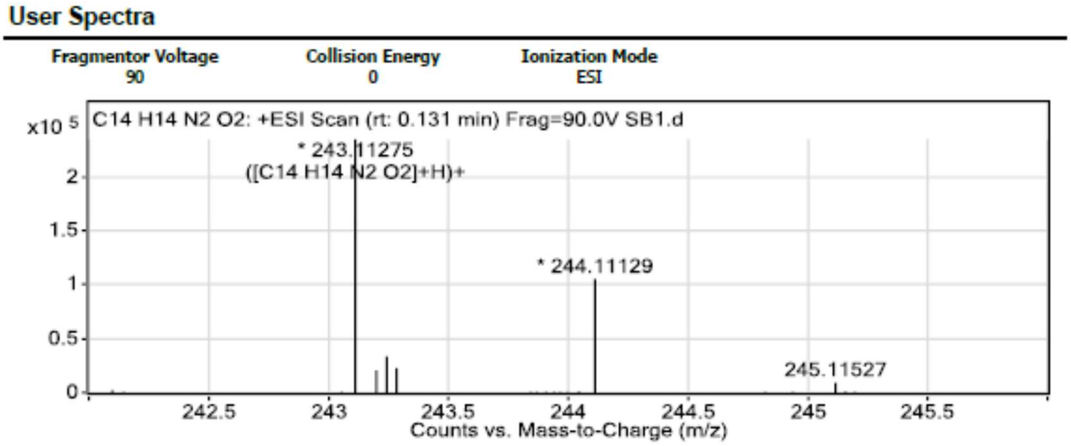

Mass spektrum of **(*E/Z*)-**5-(((4-aminophenyl)imino)methyl)-2-methoxyphenol **(14)**

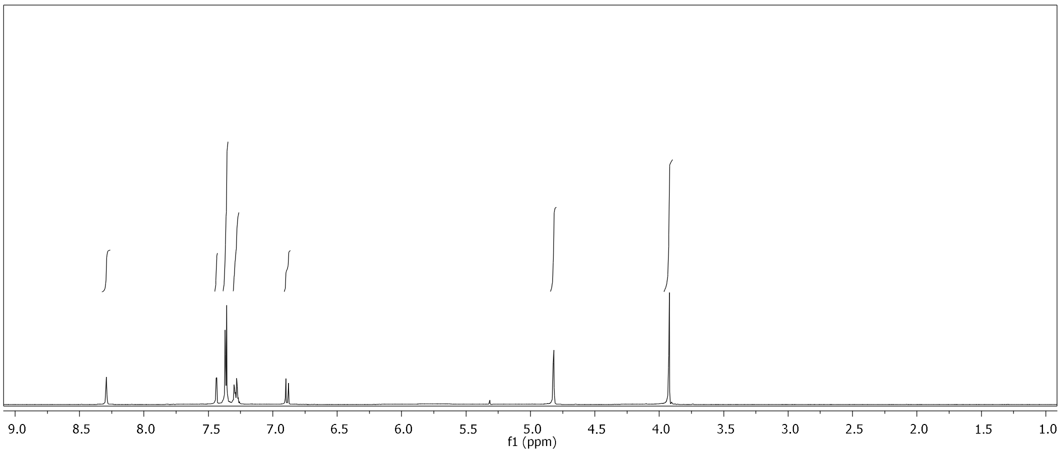

^1H NMR of 5-((benzylimino)methyl)-2-methoxyphenol **(15)** in $CDCl_3$

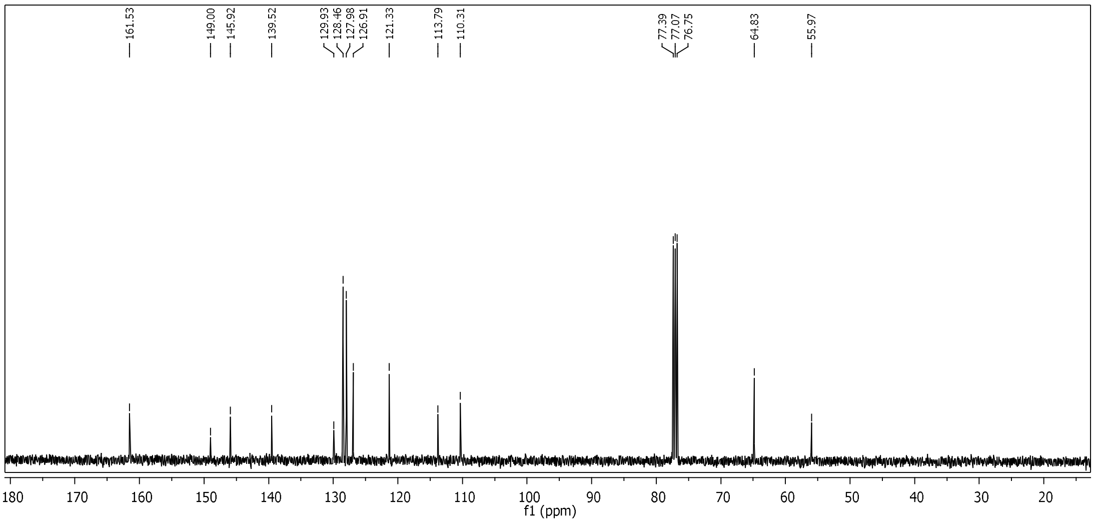

^{13}C NMR of 5-((benzylimino)methyl)-2-methoxyphenol **(15)** in $CDCl_3$

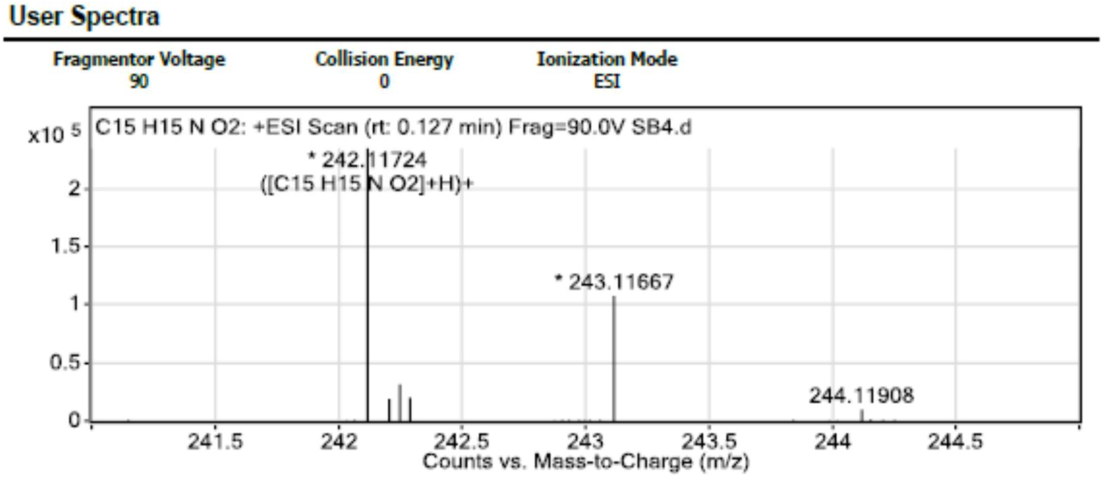

Mass spektrum of 5-((benzylimino)methyl)-2-methoxyphenol **(15)**

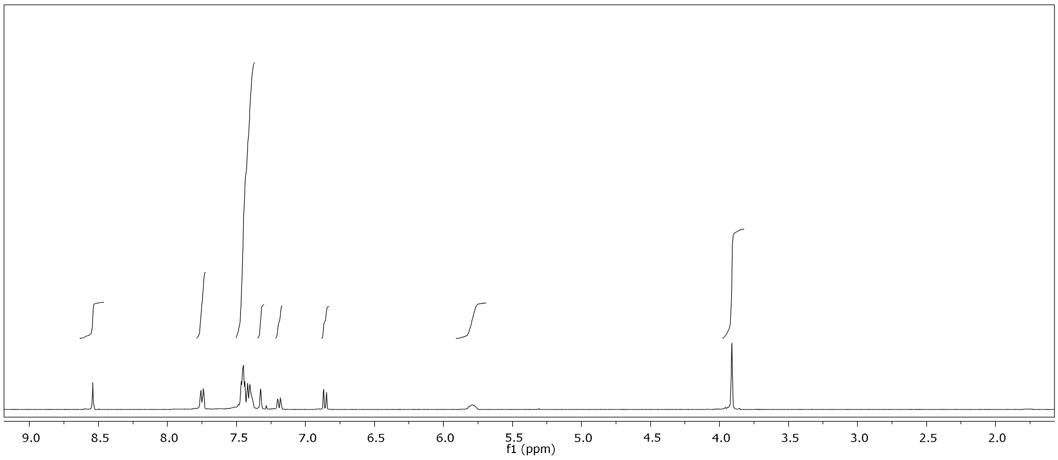

^{1}H NMR of (*E*)-5-(((diphenylmethylene)hydrazineylidene)methyl)-2-methoxyphenol (16) in $CDCl_3$

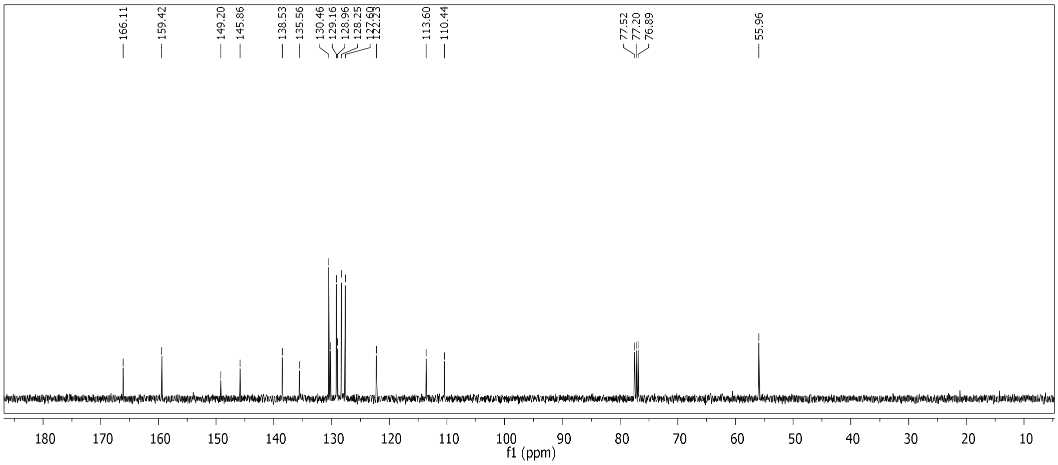

^{13}C NMR of (*E*)-5-(((diphenylmethylene)hydrazineylidene)methyl)-2-methoxyphenol (16) in $CDCl_3$

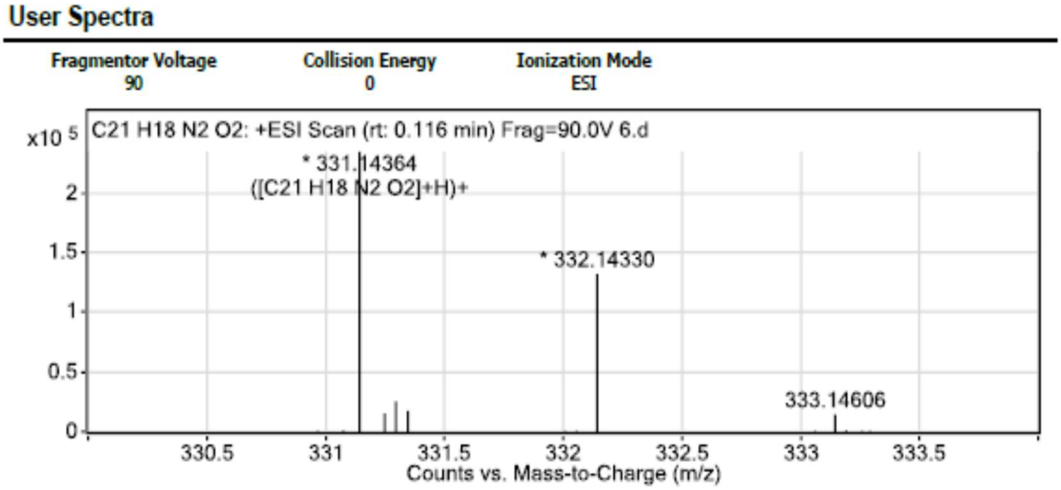

Mass spektrum of (*E*)-5-(((diphenylmethylene)hydrazineylidene)methyl)-2-methoxyphenol **(16)**

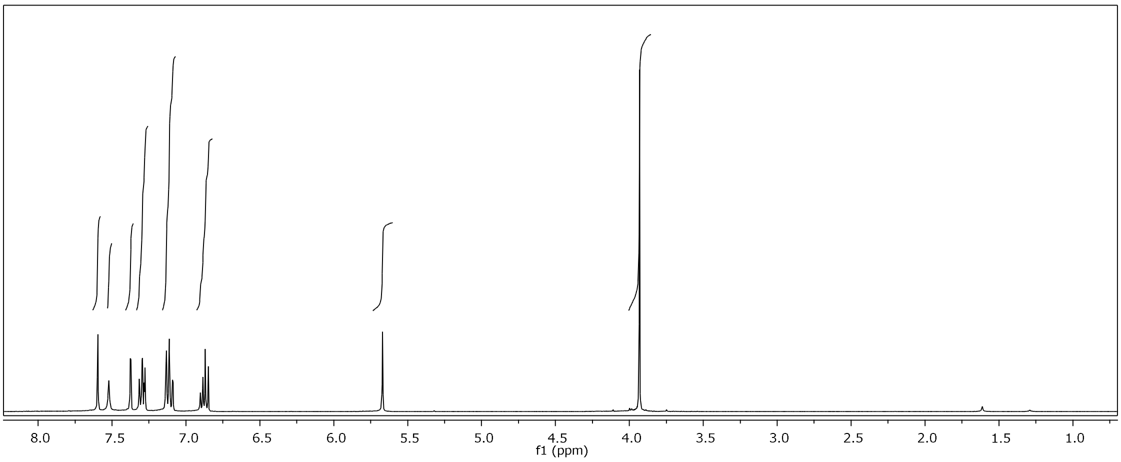

^{1}H NMR of (*E*)-2-methoxy-5-((2-phenylhydrazineylidene)methyl)phenol **(17)** in $CDCl_3$

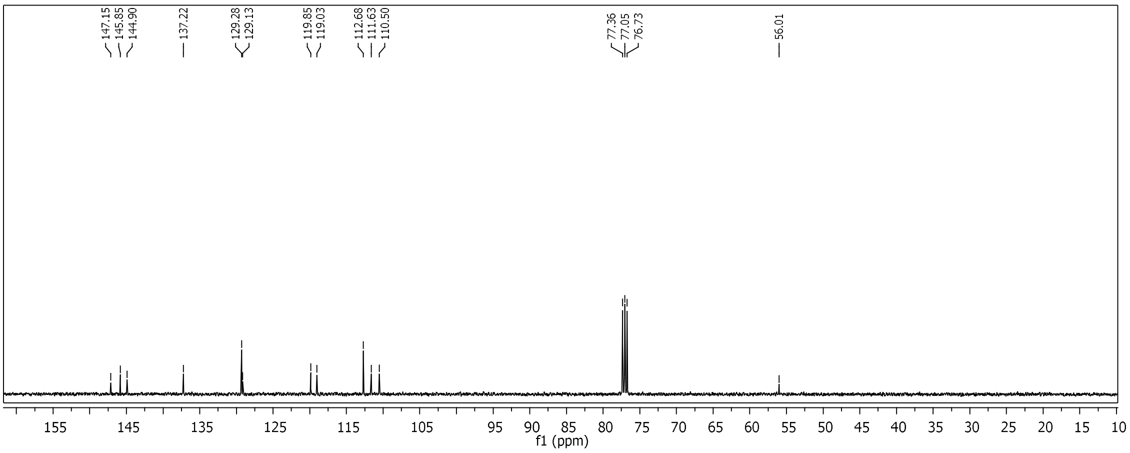

^{13}C NMR of (*E*)-2-methoxy-5-((2-phenylhydrazineylidene)methyl)phenol **(17)** in $CDCl_3$

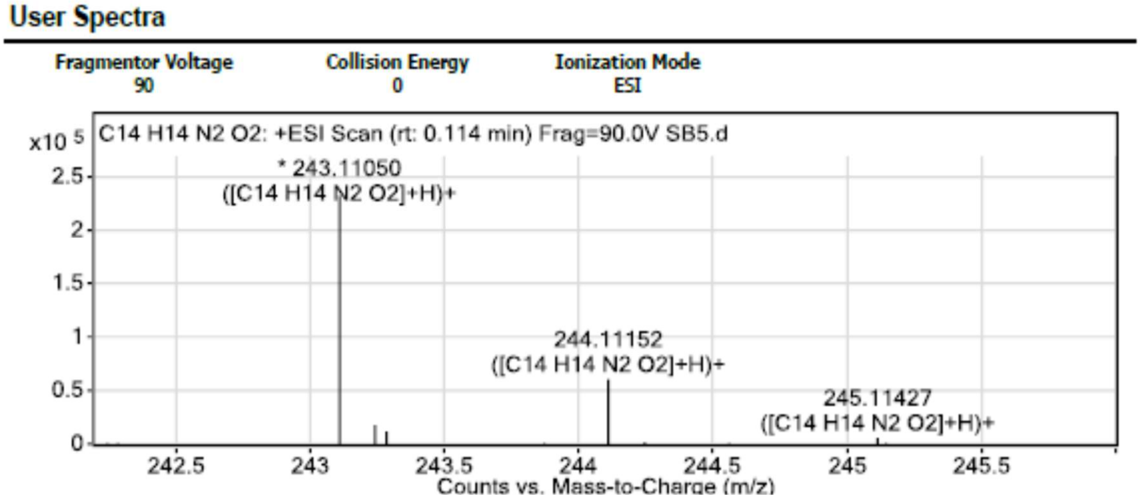

Mass spektrum of **(E)-2-methoxy-5-((2-phenylhydrazineylidene)methyl)phenol (17)**

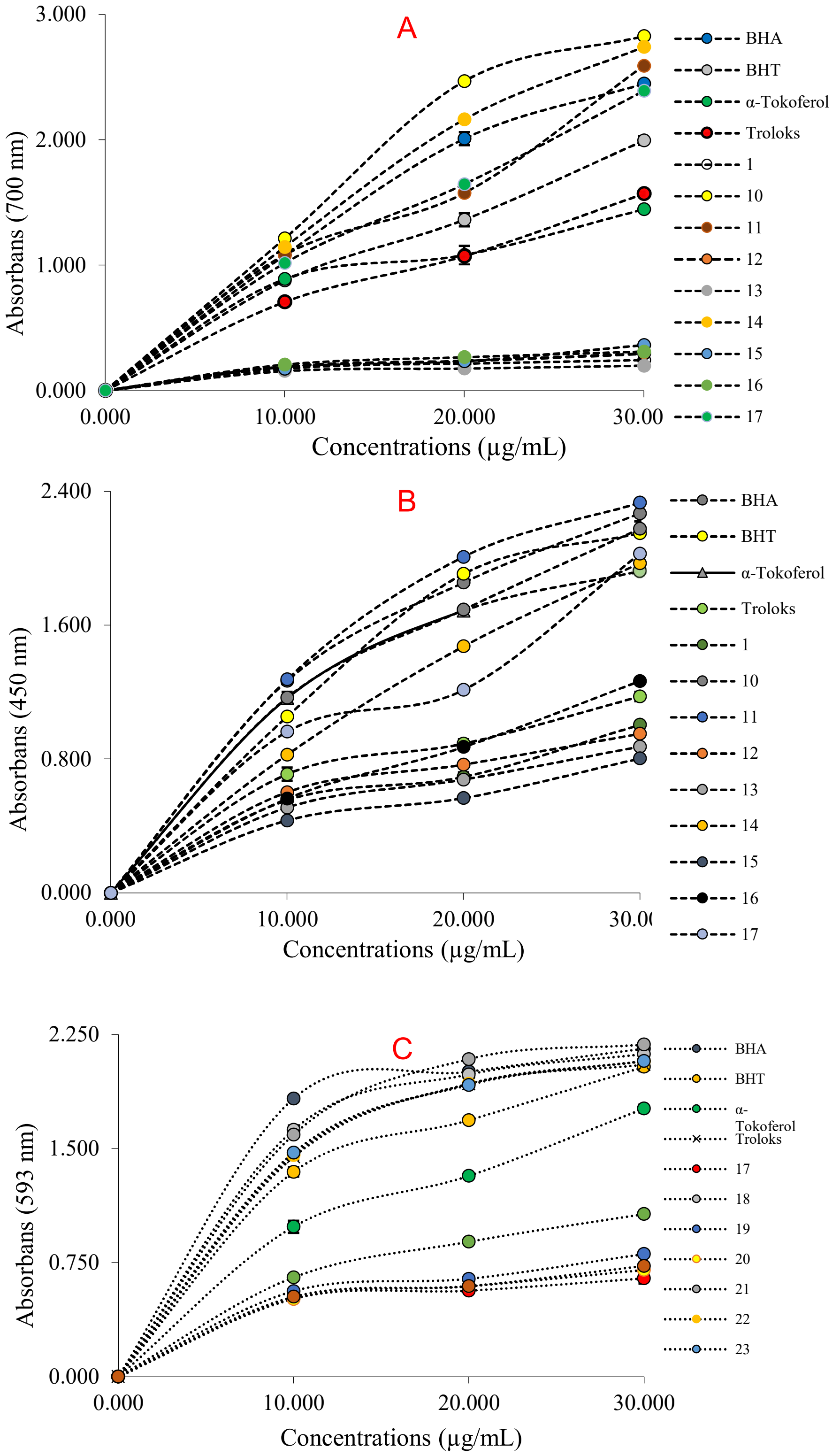

Figure S1. The reducing ability of 10-30 µg/mL of Schiff bases (**10-15**) and hydrazineylidene derivatives (**16-17**) and standards. **A**. Fe^{3+} reducing effect, **B**. Cu^{2+} reducing effect, **C**. Fe^{3+}-TPTZ reducing effect

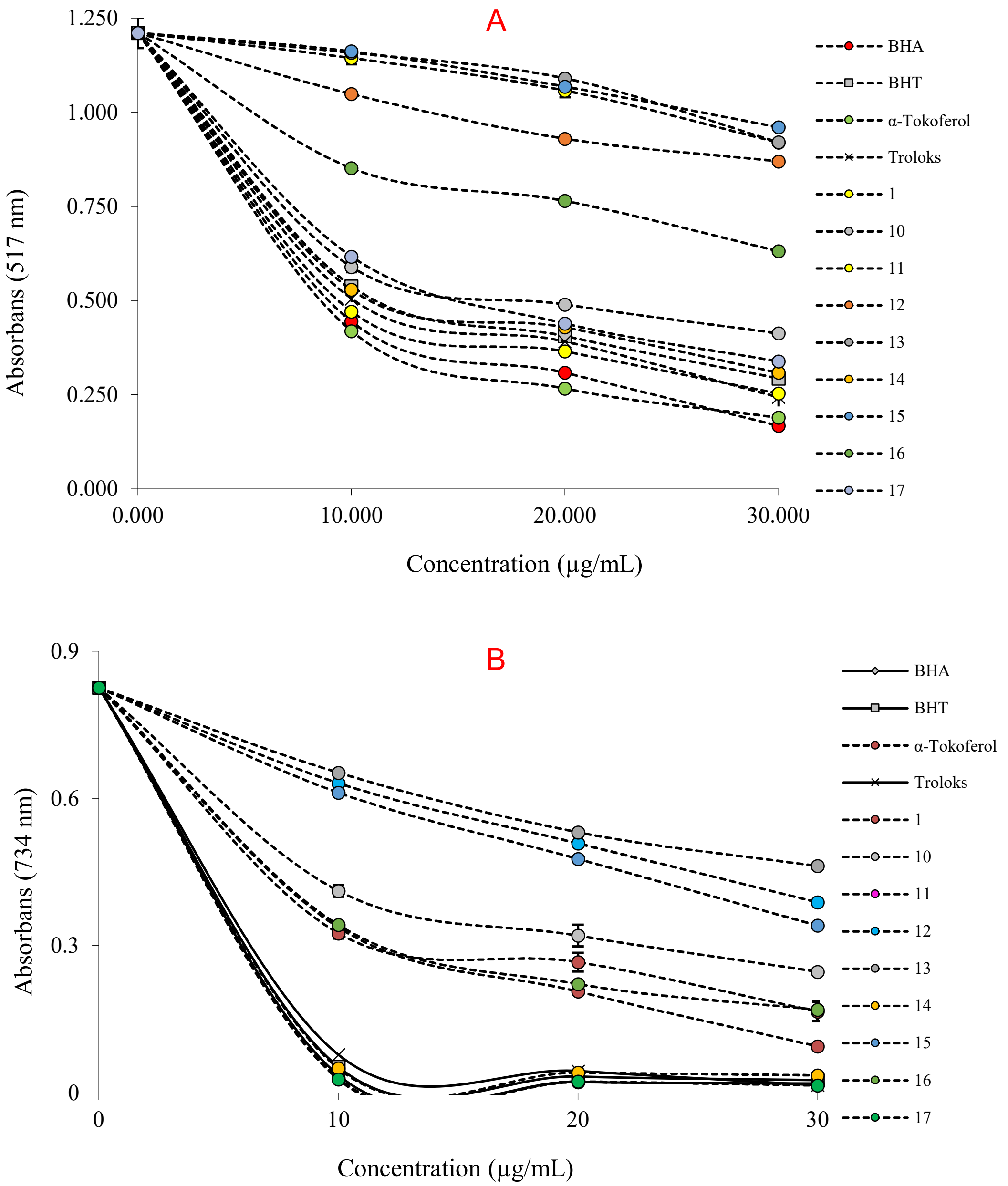

Figure 2. Radical removing abilities of different concentration (10-30 µg/mL) of Schiff bases (**10-15**) and hydrazineylidene derivatives (**16-17**) and standards. **A**. DPPH· removing effect, **B**. ABTS$^{\bullet+}$ removing effect
